# Co-treatment Strategy Supports Neuroprotection by Intersecting p62-Keap1-NRF2 and Autophagy Signaling Pathways in the Cellular Model of Parkinson's Disease

**DOI:** 10.1007/s10571-025-01610-9

**Published:** 2025-10-16

**Authors:** Oliwia Koszła, Przemysław Sołek, Krzysztof Jóźwiak

**Affiliations:** 1 Department of Biopharmacy, Medical University of Lublin, Chodźki 4a, 20-093 Lublin, Poland; 2 Department of Biochemistry and Toxicology, University of Life Sciences, Akademicka 13, 20-950 Lublin, Poland

**Keywords:** Antioxidants, Apoptosis, Autophagy, Neurodegenerative disease, Oxidative stress, Synergistic therapy

## Abstract

Parkinson’s disease (PD) is a progressive neurodegenerative disorder characterized by neuronal loss, attributed largely to oxidative stress and mitochondrial dysfunction. This study explores the synergistic neuroprotective effects of resveratrol and lithium chloride co-treatment, focusing on their impact on oxidative stress and autophagy via the p62/Keap1/Nrf2 signaling pathway. We focused on the multilevel evaluation of the monotherapy and co-treatment effects on neurons in terms of cellular and molecular features in vitro, such as metabolic activity, level of reactive oxygen/nitrogen species, total antioxidant capacity, DNA damage, level of ATP, neurite outgrowth, cell cycle and proteins level involved in intracellular signaling pathways, with particular emphasis on autophagy correlated with p62 protein. Additionally, we screened 92 genes involved in steps of protein aggregation, excitotoxicity, inflammation and oxidation. Studies have shown that co-treatment has stronger properties than monotherapy. Statistical analysis was conducted using one-way ANOVA followed by Dunnett’s post hoc test. The treatment indicated strong antioxidant properties, activation of autophagy, and the correlated apoptosis pathway via p62/Keap/Nrf2/ARE. Moreover, the therapy induced neurite outgrowth and eliminated DNA damage without disturbing the cell cycle. Finally, in the HT-22 cell line, therapy activated key genes involved in cell signaling and inflammation. In contrast, in SH-SY5Y cells, therapy engaged genes related to proteolysis, cell cycle regulation, protein kinase signaling, and lipid metabolism. These findings underscore resveratrol and lithium chloride co-treatment as a promising therapeutic strategy for mitigating oxidative damage and enhancing neuroprotection in PD. Relying on natural compounds, this combination could serve as a preventive strategy for the elderly.

**Supplementary Information:**

The online version contains supplementary material available at 10.1007/s10571-025-01610-9.

## Introduction

Parkinson’s disease (PD) is a complex and progressive neurodegenerative disorder characterized by neuronal loss. Recent studies indicate hippocampal alterations in PD, potentially leading to its atrophy (Camicioli et al. 2003; Villar-Conde et al. 2021). Although there are various causes of PD, the pathogenesis of the disease centers around oxidative stress, mitochondrial dysfunction and protein aggregation (Upadhayay et al. 2025b). The presented factors are closely related to autophagy, which is necessary for the neuronal survival and its disturbed process leads to neurodegeneration (Lynch-Day et al. 2012).

A redox imbalance between the production of reactive oxygen species (ROS) and reactive nitrogen species (RNS) and the antioxidant defense mechanism disrupts various biological processes. Excessive oxidative stress leads to DNA double-strand breaks (DSBs), cell cycle dysregulation, mitochondrial dysfunction and ultimately neuronal degeneration. Evidence suggests that mitochondrial dysfunction in PD is tightly associated with ROS overproduction, leading to a deficiency in adenosine triphosphate (ATP), crucial for proper neuronal function (Soni et al. 2024; Upadhayay et al. 2025a).

The primary signaling axis acting as a defense system against oxidative stress is the nuclear factor erythroid 2-related factor 2 (Nrf2)–Kelch-like ECH-associated protein 1 (Keap1)–antioxidant response element (ARE) pathway (Nrf2/Keap1/ARE) (Kim et al. 2020). Nrf2 is a transcription factor that protects cells from oxidative stress. In the cytoplasm, Nrf2 interacts with Keap1, an oxidation–reduction sensor that reduces ROS levels. High levels of free radicals disrupt the Nrf2/Keap1 interaction, allowing Nrf2 to translocate to the nucleus. In the nucleus, Nrf2 binds to ARE, initiating the expression of cytoprotective genes (Yang et al. 2022). This is the so-called canonical mechanism. An alternative regulation of Nrf2 involves the p62 autophagy-related protein (sequestosome 1-SQSTM1) in a Keap1-dependent manner. Also called the non-canonical mechanism, p62 may compete with Nrf2 for Keap1 binding. As a consequence, p62 sequesters Keap1 to the autophagosome, preventing Keap1-mediated degradation of Nrf2. Dysfunction in autophagy leads to prolonged activation of Nrf2, potentially contributing to Parkinson’s disease (Jiang et al. 2015). Current treatments for PD primarily alleviate symptoms without halting disease progression or reversing brain changes, underscoring the need for a deeper understanding of PD pathomechanisms and the development of effective therapies. Recent research has focused on identifying compounds with neuroprotective properties, particularly those enhancing antioxidant defense. Resveratrol, a compound found in many plants, has shown significant neuroprotective potential due to its strong antioxidant properties (dos Santos et al. 2022). Moreover, over the last decades, lithium chloride has been proven to be characterized as a potential therapeutic agent for the treatment of neurodegenerative diseases. The mechanism of action of lithium is not entirely clear, but it also focuses on reducing oxidative stress in cells and strengthening neurotrophic factors (Lazzara and Kim 2015).

Our study investigated the unique co-treatment with natural substances, resveratrol and lithium, providing evidence that combined treatment is more effective than monotherapy. This approach targets intersecting pathways of oxidative stress and autophagy via p62/Keap1/Nrf2 signaling. Co-treatment significantly protects cells against oxidative stress, reduces DNA damage, maintains cell cycle integrity and supports neurite outgrowth. This combination could advance the development of more effective therapies and serve as a preventive strategy in the elderly. To our knowledge, the combination of resveratrol and lithium in this context was used for the first time.

## Methods

### Cell Culture

The HT-22 mouse hippocampal neuronal cell line (Merck, SCC129, RRID) and the SH-SY5Y human neuroblastoma cell line (ATCC, CRL-2266, RRID) were cultured in DMEM (Corning, USA) and DMEM/F-12 (50/50, v/v%) (Corning, USA), respectively. Both media were supplemented with 10% fetal bovine serum (FBS) (Gibco, USA) and an antibiotic mix (100 U/mL penicillin, 0.1 mg/mL streptomycin) (Thermo Fisher Scientific, USA). HT-22 cells were seeded at a density of 3.0 × 10^3^ cells/cm^2^ and SH-SY5Y cells at 3.0 × 10^4^ cells/cm^2^. Cultures were maintained in an incubator (New Brunswick Galaxy 170R, Thermo Fisher Scientific, USA) at 37 °C with 5% CO₂ to ensure physiological pH. To ensure the reproducibility and validity of our results, all experiments were performed using cell cultures maintained within a passage range of 3 to 10, thereby avoiding potential phenotypic changes associated with prolonged subculturing.

### Compounds and Treatment

Resveratrol (Res), lithium chloride (LiCl) and 6-hydroxydopamine hydrochloride (6-OHDA) (Sigma-Aldrich, Germany) were prepared according to the manufacturer's instructions. 6-OHDA, used to induce an in vitro model of Parkinson’s disease, served as a positive control. Specifically, Res and 6-OHDA were dissolved in DMSO to create 100 mM stock solutions, while LiCl was prepared directly in the culture medium to a 1 M stock solution. Prior to experimentation, stock solutions were diluted in complete medium (DMEM or DMEM/F-12) and administered for a 48-h incubation.

### MTT Assay

MTT assay was performed as described elsewhere (Solek et al. 2019). Cells were seeded at standard densities in 96-well plates and treated with compounds at selected concentrations. After 48 h of incubation, MTT (Sigma-Aldrich, Germany) was added to a final concentration of 5 mg/mL for 4 h, followed by the addition of 150 µL of DMSO. Absorbance was measured at 590 nm with a reference at 620 nm using a BioTek Synergy H1 microplate reader (Agilent, USA).

### Cellular Redox Homeostasis Analysis

The assay was performed as described previously (Koszła et al. 2022). Both cell lines were seeded at standard densities in black, flat-bottom 96-well plates, treated with Res, LiCl or 6-OHDA at selected concentrations, and probed with dihydroethidium for superoxide, DAF-2 diacetate for nitric oxide (Cayman Chemical, USA) and thiol tracker violet for reduced glutathione (GSH) (Thermo Fisher Scientific, USA) at a final concentration of 5 µM each. Digital images and quantifications were obtained using a Pathway 855 microscopic fluorescence imaging system (Becton Dickinson, USA) and expressed as relative fluorescence units (RFU).

### Total Antioxidant Capacity Analysis

The antioxidant assay was performed according to the manufacturer’s recommendations (Sigma-Aldrich, Germany). HT-22 cells were seeded at a density of 3.0 × 10^3^ cells/cm^2^ and SH-SY5Y cells at 3.0 × 10^4^ cells/cm^2^ in 96-well plates, treated with compounds and incubated for 48 h. Following treatment, myoglobin and ABTS substrate working solutions were added. Plates were incubated at room temperature for 5 min, followed by the addition of 100 μL of stop solution. Absorbance was measured at 405 nm using a BioTek Synergy H1 microplate reader (Agilent, USA).

### Activity of the Nrf2 Antioxidant Pathway

HT-22 and SH-SY5Y cells were seeded in 96-well plates at standard densities. After 24 h, cells were transfected with a transfection-ready ARE luciferase reporter vector and a non-inducible firefly luciferase vector in Opti-MEM I medium (antibiotic-free) using Lipofectamine 2000 (Thermo Fisher Scientific, USA). Following overnight incubation, cells were treated with compounds for 48 h. Luciferase activities were measured using the dual luciferase (firefly-renilla) assay system (Biosciences, USA) according to the manufacturer’s protocol on a Synergy H1 microplate reader (Agilent, USA).

### Neurite Outgrowth and Cell Viability Measurement

Neurite outgrowth was assessed using the Molecular Probes neurite outgrowth staining kit according to the manufacturer’s recommendations (Thermo Fisher Scientific, USA). Cells were seeded and treated with compounds, incubated for 48 h and then stained with the working stain solution for 20 min at room temperature. After rinsing with DPBS and applying the background suppression solution, fluorescence was measured using the Pathway 855 microscopic fluorescence imaging system (Becton Dickinson, USA).

### DNA Damage and Cytotoxicity Analysis

DNA damage was assessed using the HCS DNA damage kit according to the manufacturer’s recommendations (Thermo Fisher Scientific, USA). HT-22 and SH-SY5Y cells were seeded in black 96-well plates, treated with compounds and incubated for 48 h. Cells were stained with the Image-iT Dead Green viability stain working solution for 30 min at 37 °C, fixed with 4% paraformaldehyde for 15 min, permeabilized with Triton X-100 in PBS for 15 min and blocked with 1% BSA in PBS for 60 min. Primary antibody solution was added for 60 min at room temperature, followed by secondary antibody/counterstain solution for another 60 min. Plates were protected from light, rinsed with DPBS and imaged using the Pathway 855 microscopic fluorescence imaging system (Becton Dickinson, USA).

### Cell Cycle Profile

Cells were seeded in black 96-well plates, treated with compounds for 48 h and fixed in 3.7% formaldehyde for 20 min at room temperature. Cells were then stained with Hoechst 33258 at 1 µg/mL for 10 min, washed with DPBS,and imaged using the Pathway 855 microscopic fluorescence imaging system (Becton Dickinson, USA). Cell cycle profiles were analyzed using ImageJ software with the DNA Cell Cycle plug-in, presenting results as percentages of G0/G1, S and G2/M phases of total cell count.

### Protein Quantification by Western Blot

The procedure was previously described by (Koszła et al. 2020). Briefly, cells were seeded in T75 culture flasks at standard densities and treated with compounds for 48 h. Total protein extracts were isolated using RIPA buffer supplemented with 1 mM PMSF (Thermo Fisher Scientific, USA), homogenized for 45 min at 4 °C and centrifuged at 15,000×*g* for 15 min. Supernatants were collected and protein concentration was determined using the BCA method (Thermo Fisher Scientific, USA) with bovine serum albumin (BSA) as a standard. Samples (10 µg) were prepared in Laemmli buffer, resolved by 10% SDS-PAGE and transferred onto PVDF membranes. Membranes were blocked with 1% BSA in TBST for 60 min at room temperature, incubated overnight at 4 °C with primary antibodies in blocking buffer, washed with TBST and incubated with HRP-conjugated secondary antibodies in blocking buffer for 1 h at room temperature. Signal detection was performed using the ECL method (Westar Supernova, Cyanagen, Italy) and imaged with an Azure c400 system (Azure Biosystems, USA).

### Real-Time PCR-Based Gene Expression Profiling

Total RNA from both cell cultures was isolated using TRIzol reagent (Sigma-Aldrich, USA) according to the manufacturer’s protocol. RNA concentration and purity were then determined by measuring absorbance at 260 nm and 280 nm using a NanoDrop spectrophotometer.

The High-Capacity cDNA Reverse Transcription Kit was applied for quantitative conversion of 2 μg of total RNA into single-stranded cDNA following the manufacturer’s protocol (Thermo Fisher Scientific, USA). The resulting cDNA was used as a template for subsequent quantitative real-time PCR (qRT-PCR) analyses to evaluate gene expression levels.

For real-time PCR, the Applied Biosystems TaqMan Array Human Alzheimer’s Disease Panel was used (Thermo Fisher Scientific, USA). The plates contained 92 assays targeting Alzheimer’s-associated genes and 4 assays for candidate endogenous control genes. Each reaction sample contained 10 ng of cDNA, while the negative control contained sterile water. The experiment was normalized to the reference gene, which served as the internal control. Product growth analysis was performed by measuring the fluorescence of the TaqMan probe using the StepOnePlus Real-Time PCR System (Applied Biosystems). Gene expression analysis was conducted using the comparative $2^{{ - \Delta \Delta C_{{\text{t}}} }}$ method, calculating the difference in expression levels between the test samples and the reference sample.

### Statistics

The test groups were compared to the untreated control and the raw data were analyzed by one-way analysis of variance ANOVA followed by Dunnett multiple comparisons post hoc test in GraphPad Prism 6.0. The results are presented as mean ± standard deviation of at least three independent experiments (*n* = 3). The differences to the control group are marked as *, while the comparison to 6-OHDA as ^#^.  ^.^*P* < 0.05 was considered statistically significant and displayed as *^/#^*P* < 0.05; **^/##^*P* < 0.01; ***^/###^ P < 0.001 . * Indicates the comparison between the control and positive control with Parkinson’s model disease (6-OHDA), # indicates the comparison between positive control (6-OHDA) and treatment group with Res, LiCl, Res + LiCl.

## Results

### Increased Oxidative Stress Contributes to Low Cell Metabolic Activity

**Fig. 1:**
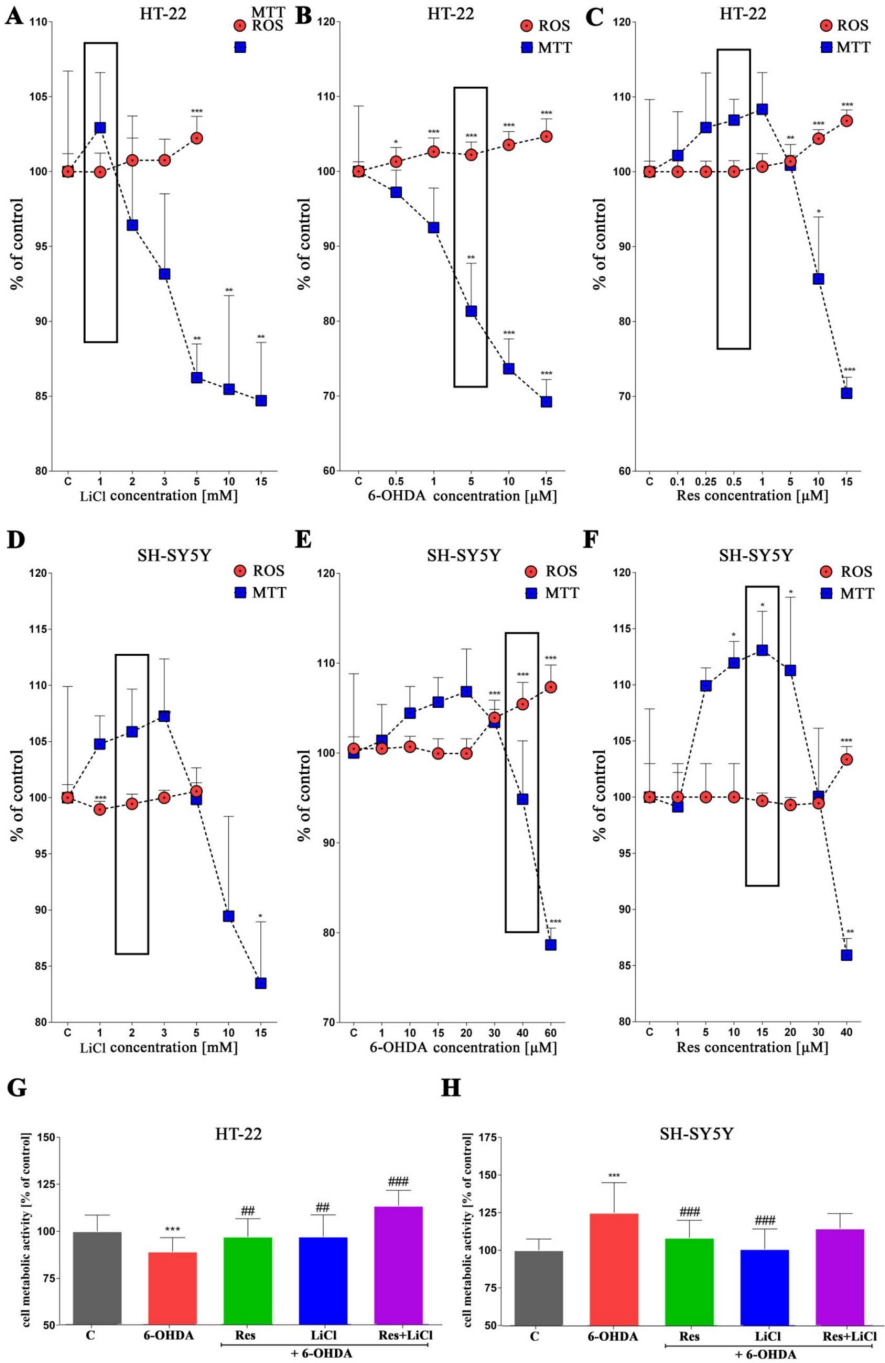
Dose-dependent effects of Res, LiCl and Res + LiCl on HT-22 and SH-SY5Y cells in terms of general homeostasis. Metabolic activity assessed by MTS assay (**A**–**H**) and reactive oxygen species levels measured by dihydroethidium probe (**A**–**F**). Concentrations selected for further investigation are marked with black frames

In the beginning, we observed dose-dependent changes in the cell metabolic activity. Specifically, we noted increased metabolic activity in most experimental sets at lower concentrations of the substances tested. Conversely, higher concentrations resulted in significant decreases in metabolic activity. This observation was consistent across all sets, regardless of the cell line studied. Interestingly, HT-22 cells appeared to be more sensitive to the substances tested, as evidenced by lower viability than the control. Additionally, increasing concentrations led to oxidative stress upregulation (Fig. 1A–F). The lowest viability and, at the same time, highest oxidative stress were observed for 6-OHDA. These results confirmed the induction of the PD model by 6-OHDA (Fig. 1B, E). Concentrations marked with a black frame were selected for further studies. For resveratrol and lithium chloride, concentrations that caused an increase in cell metabolic activity and a decrease in oxidative stress were chosen. In turn, for 6-OHDA, the concentration that increased reactive oxygen species and decreased cell viability was selected to effectively induce the PD model.

Subsequently, in the established experimental sets with the PD model treated with monotherapy and co-therapy, cell metabolic activity was examined. In healthy neuronal cells, an increase in viability was observed for Res, LiCl or co-treated. For the positive control with the PD model (6-OHDA), the expected decrease in metabolic activity was observed (Fig. 1G). Furthermore, the observed activities were reversed in the case of neuronal cancer cells. Only for the PD control, an increase in metabolic activity was observed, while for the treated sets, a decrease was noted (Fig. 1H).

### Elimination of ROS/RNA Overproduction By Antioxidant Activity

**Fig. 2:**
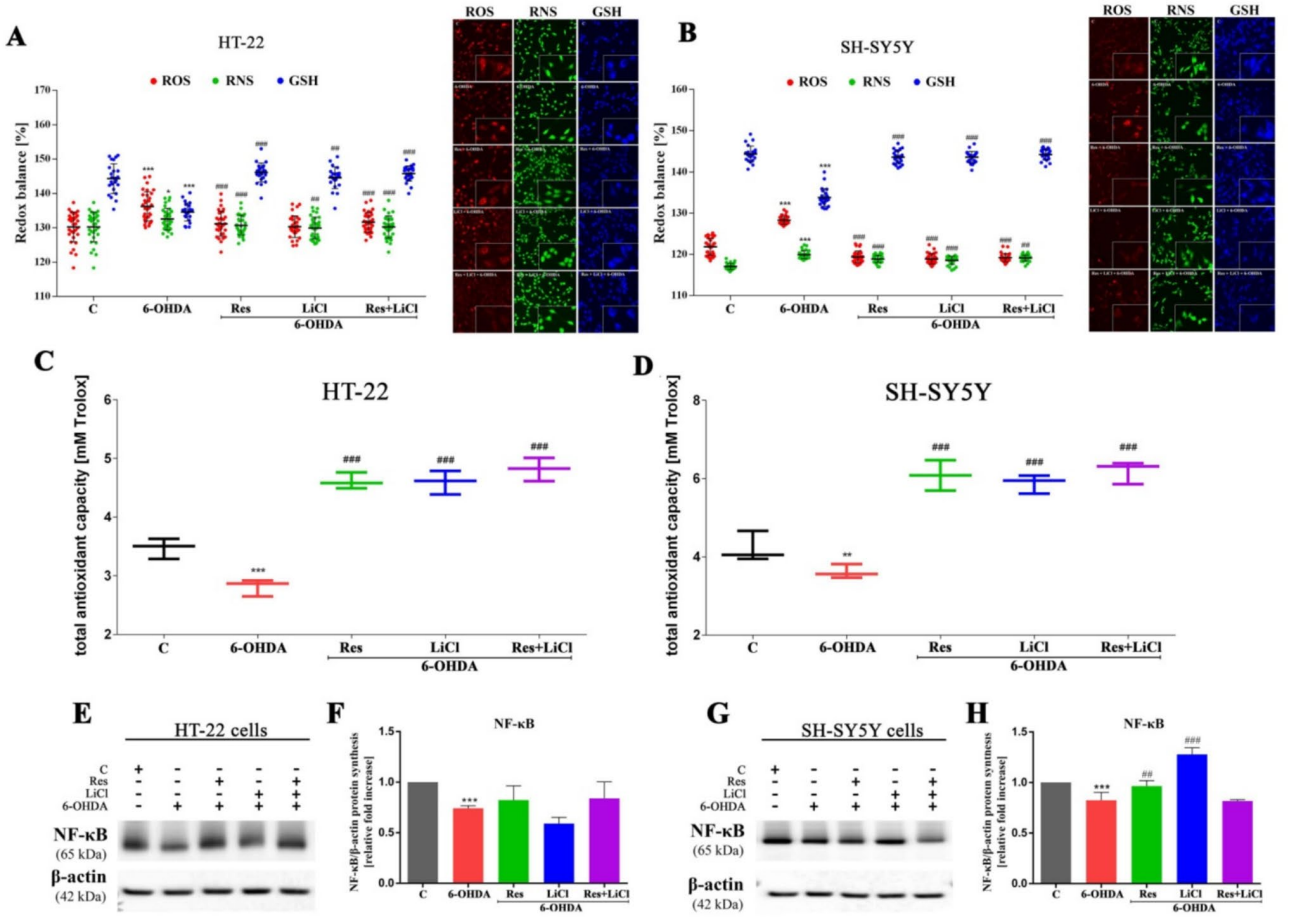
Modulation of cellular redox status in HT-22 and SH-SY5Y cells by Res, LiCl and Res + LiCl. The parameters measured include reactive oxygen species, reactive nitrogen species and reduced glutathione (**A**, **B**), along with total antioxidant capacity (**C**, **D**) and NF-κB regulation (**F**, **H**). Representative immunoblots are presented (**E**, **G**)

Further, the PD model was characterized by elevated ROS and RNS production, alongside a decreased GSH pool, in comparison to the control group (C). Conversely, treatment with either monotherapy or combination therapy resulted in a reduction of ROS and RNS levels and an increase in GSH production. This pattern was consistently observed across both cell lines examined (Fig. 2A, B). Furthermore, we observed an improved total antioxidant capacity in sets treated with resveratrol and lithium, as well as in the co-treatment group, while the PD control model expressed a significant decrease in antioxidant defense status. This observation was consistent for both cell lines (Fig. 2C, D).

At the molecular level, the expression of the NF-κB transcription factor role in altered ROS production was assessed (Fig. 2F, H). In the PD model, downregulation of NF-κB protein was observed. Additionally, no significant changes were noted for any experimental sets in the case of the HT-22 cell line (Fig. 2E, F). However, upregulation was noted only for resveratrol and lithium chloride monotherapy compared to 6-ODHA for the SH-SY5Y cell line (Fig. 2G, H).

### Activation of the p62–Keap1–NRF2 Pathway to Alleviate Oxidative/Nitrosative Stress

**Fig.3:**
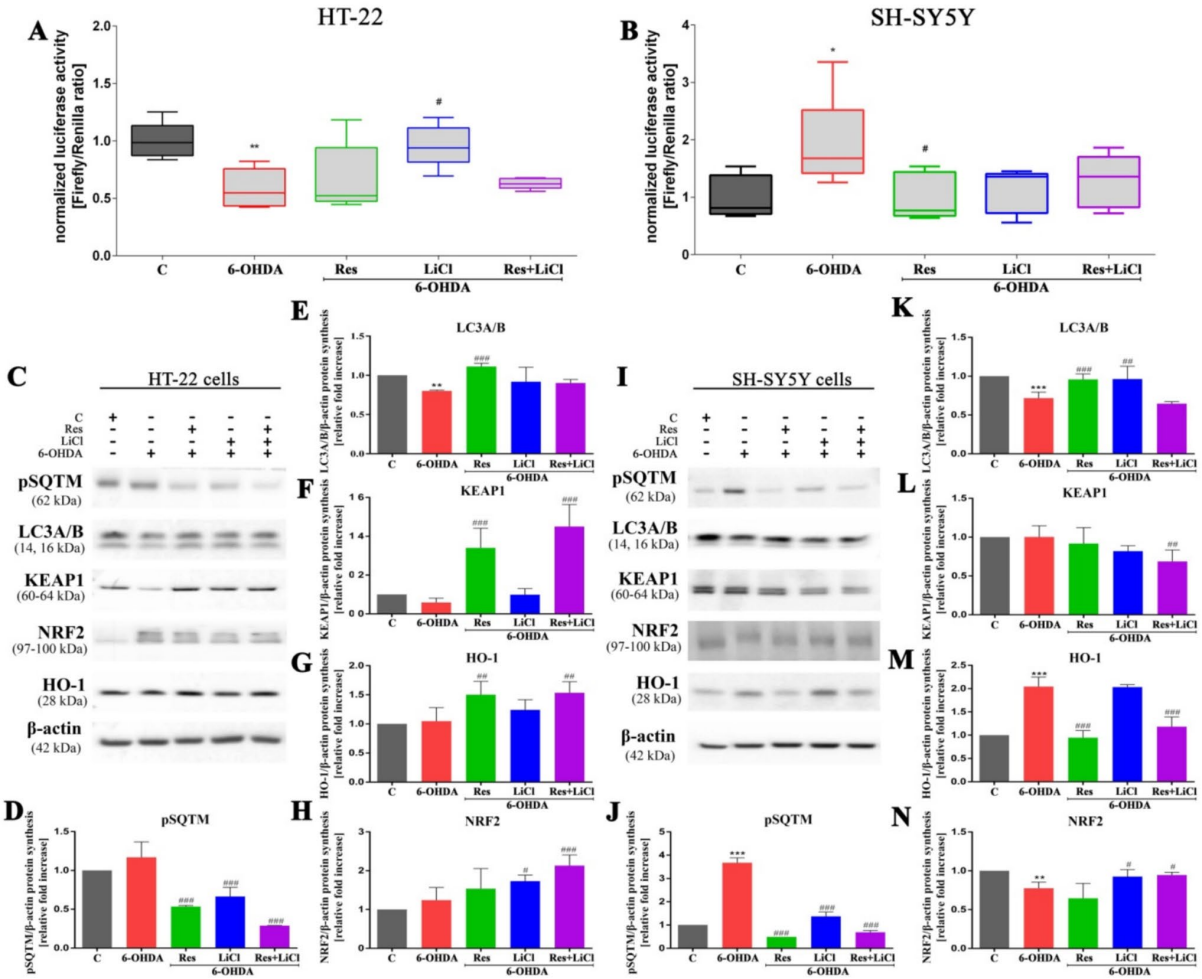
Signal modulation across p62/Keap1/NRF2/ARE antioxidant response pathway HT-22 and SH-SY5Y cells by Res, LiCl and Res + LiCl. Antioxidant response element (ARE) measurement (**A**, **B**) and Western Blot densitometry analysis of pSQTM (**D**, **J**), LC3A/B (**E**, **K**), Keap1 (**F**, **L**), HO-1 (**G**, **M**), Nrf2 (**H**, **N**) protein synthesis levels. Representative immunoblots are presented (**C**, **I**)

At the next stage, the activity of the antioxidant response element (ARE), a critical component of the p62/Nrf2/Keap1/ARE pathway, was analyzed. In the HT-22 cell line, a decrease in ARE activity was observed in the control PD model, while an increase in ARE activity was noted in the LiCl-treated sets (Fig. 3A). In turn, in the SH-SY5Y cell line, the effects were reversed. Specifically, the positive control demonstrated an upregulation, whereas the Res-treated group exhibited a reduction in ARE activity (Fig. 3B).

Subsequently, an analysis of the expression of proteins associated with the Nrf2-based pathway was performed (Fig. 3C, I). In the HT-22 cell line, the PD model, characterized by reduced ARE activity, showed no activation of the Nrf2 pathway as anticipated. Specifically, we observed no changes in the levels of pSQTM (Fig. 3D), KEAP1 (Fig. 3F), HO-1 (Fig. 3G), or NRF2 (Fig. 3H), with the exception of a decrease in LC3A/B expression (Fig. 3E). In contrast, treatment produced more changes in expression. All experimental groups exhibited a significant decrease in pSQTM expression, regardless of the treatment. Additionally, a notable decrease in KEAP1 expression was observed only in the combination treatment group. On the contrary, an increase in LC3A/B expression was noted in the Res group, for HO-1 in Res and co-treatment groups and for NRF2 in the LiCl and co-treatment groups (Fig. 3E–H). In the positive control PD model for the SH-SY5Y cell line, an elevation in antioxidant response element activity was accompanied by the up-regulation of proteins associated with the p62/Nrf2/Keap1 signaling pathway. In detail, we observed increased expression of pSQTM (Fig. 3J) and HO-1 (Fig. 3M), but a decrease in LC3A/B and NRF2. In all treatment groups, pSQTM expression decreased, while HO-1 expression decreased in the Res and combined groups only. In contrast, increased expression of LC3A/B (Fig. 3K) was observed in the Res and LiCl monotherapy groups, KEAP1 expression increased in the Res and combined groups (Fig. 3L) and NRF2 expression increased in the LiCl and combined set (Fig. 3N). In summary, the co-treatment set demonstrated the highest overall activation of proteins within this pathway and this trend was consistent across both cell lines tested.

### p62 Mediated Activation of Autophagy

**Fig. 4:**
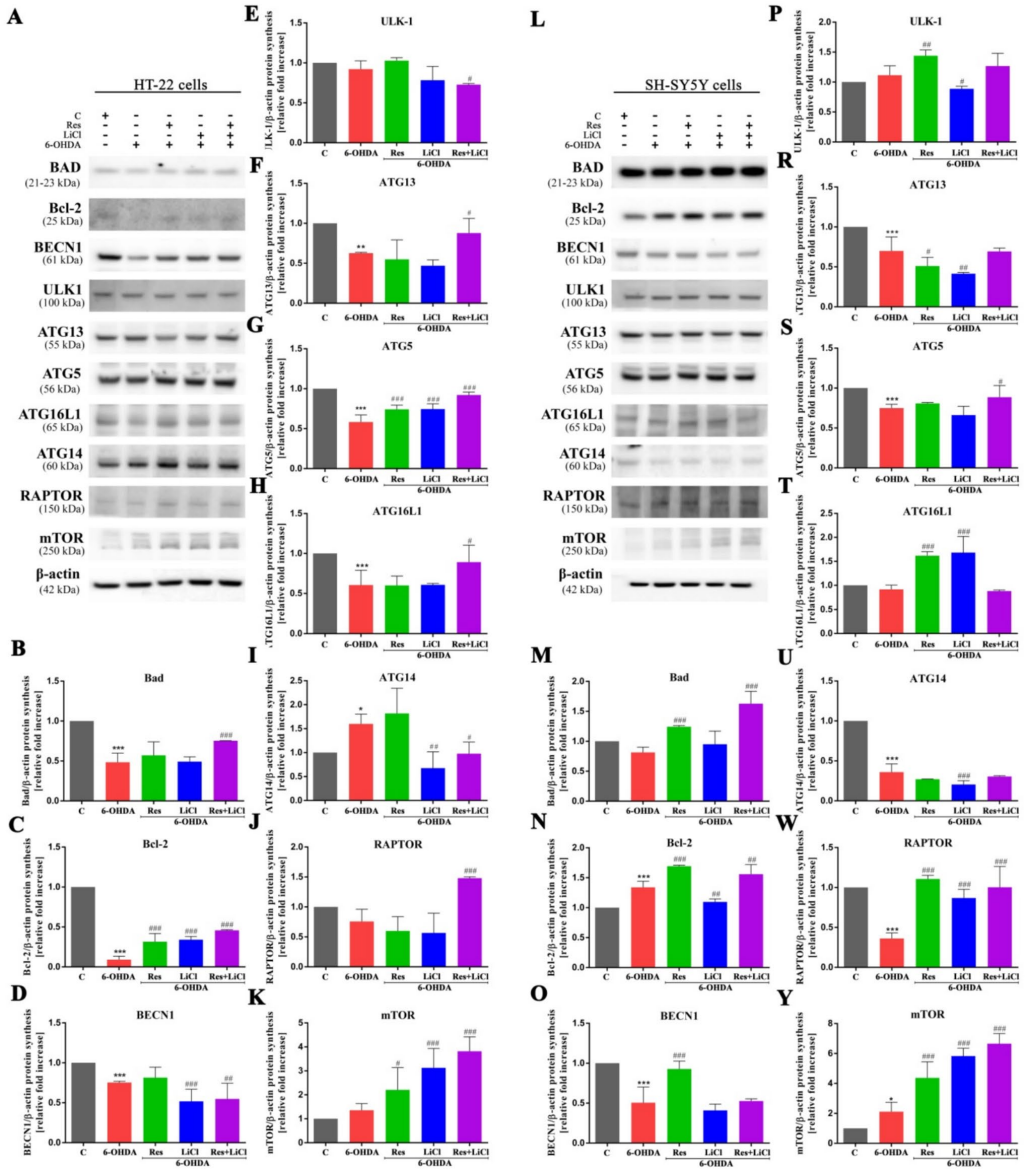
Cell-specific stimulation of the cellular autophagy pathway in HT-22 and SH-SY5Y cells by Res, LiCl and Res + LiCl. Western Blot densitometry analysis of Bad (B, M), Bcl-2 (C, N), BECN1 (D, O), ULK-1(E, P), ATG13 (F, R), ATG5 (G, S), ATG16L1 (H, T), ATG14 (I, U), RAPTOR (J, W), mTOR (K, Y) protein synthesis levels. Representative immunoblots are presented (A, L)

Given that the Nrf2 pathway can stimulate autophagy through p62, we investigated the proteins involved in this mechanism (Fig. 4A, L). In the HT-22 cell line within the PD model, we observed downregulation of Bad (Fig. 4B) and Bcl-2 (Fig. 4C), which are responsible for apoptosis activation. Additionally, there was a notable depletion of BECN1 (Fig. 4D), which, when complexed with Bcl-2, inhibits autophagy. We also noted decreases in the expression of ATG13 (Fig. 4F), ATG5 (Fig. 4G) and ATG16L1 (Fig. 4H), whereas there was no activation of the autophagy-regulating complex proteins RAPTOR (Fig. 4J) and mTOR (Fig. 4K). UKL-1 (Fig. 4E) expression remained unchanged. Interestingly, only ATG14 showed increased expression in this group (Fig. 4I). In the therapeutic groups, the trend was completely reversed. Treatment led to increased expression of Bad, ATG13, ATG16L1 or RAPTOR in the combined group, and for Bcl-2, ATG5 or mTOR across all treatment groups. Conversely, reductions were observed for BECN1 and ATG14 in the LiCl and combined groups and UKL-1 in the combined group only.

In the SH-SY5Y cell line PD model, no changes in the expression of Bad (Fig. 4M), UKL-1 (Fig. 4P), or ATG16L1 (Fig. 4T) were observed. Upregulation was noted for Bcl-2 (Fig. 4N) and mTOR (Fig. 4Y), while downregulation was observed for BECN1 (Fig. 4O), ATG13 (Fig. 4R), ATG5 (Fig. 4S), ATG14 (Fig. 4U) and RAPTOR (Fig. 4W). Similar to the HT-22 cell line, numerous changes were observed in the therapeutic groups. Specifically, in the Res and combined treatment, upregulation of proteins involved in apoptosis and autophagy, including Bad, Bcl-2, BECN1, ULK-1, ATG16L1 (only Res), ATG5 (only combination), RAPTOR and mTOR, was observed. In some cases, LiCl monotherapy also resulted in increased ATG16L1, RAPTOR and mTOR expression. Overall, downregulation was less frequent and primarily observed in the LiCl monotherapy groups, specifically for Bcl-2 (LiCl), ULK-1 (LiCl), ATG13 (Res and LiCl) and ATG14 (LiCl). In summary, the combined treatment demonstrated the highest overall activation of autophagy-related proteins, with consistent trends observed across both HT-22 and SH-SY5Y cell lines.

### Induction of Autophagy Supports the Rearrangement of Cellular Function

**Fig. 5:**
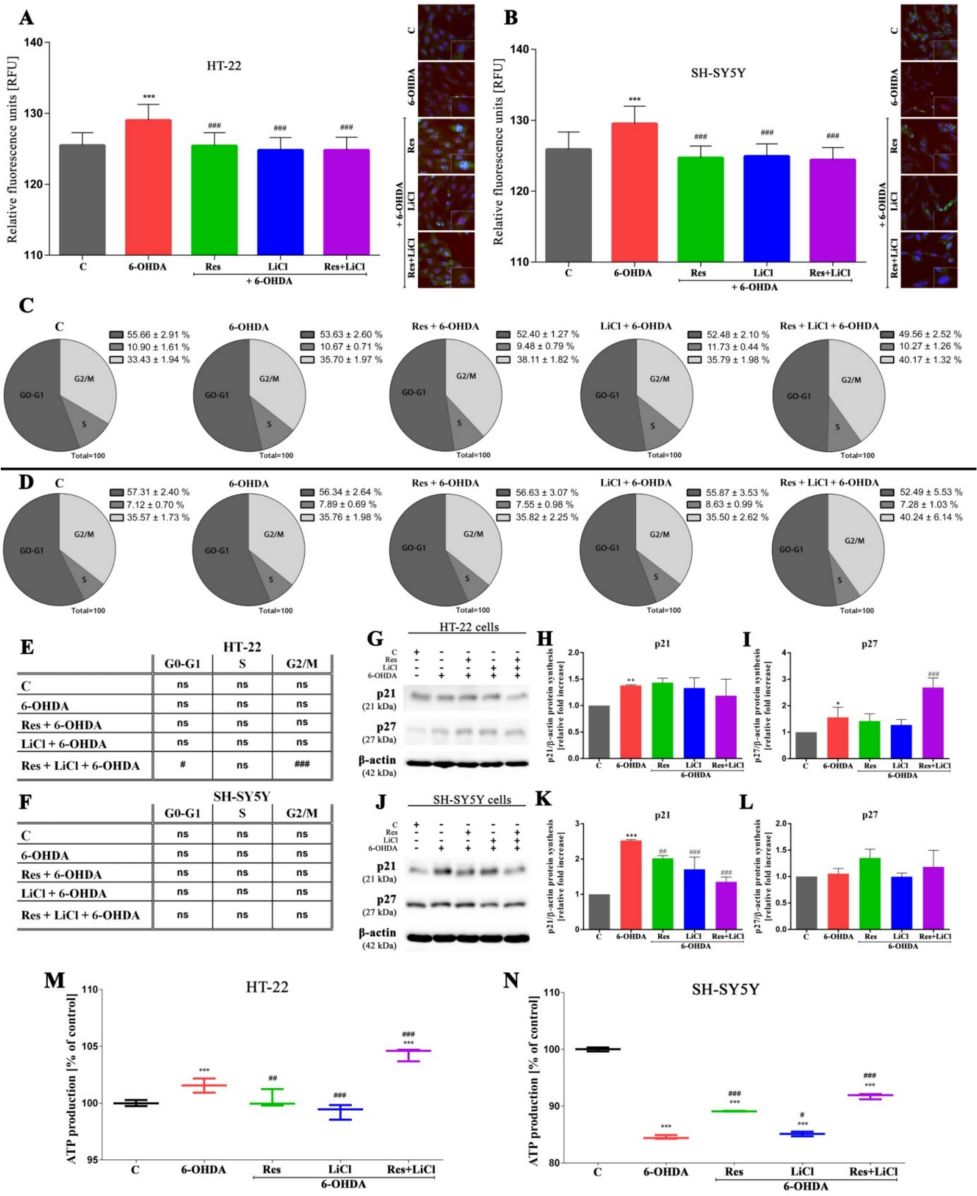
Alteration of cellular activities in HT-22 and SH-SY5Y cells by Res, LiCl, Res + LiCl in terms of DNA damage (**A**, **B**), cell cycle profile (**C**–**F**) with p21, p27 protein expression (**H**, **I**, **K**, **L**) and ATP production (**M**–**N**). Representative immunoblots are presented (**G**, **J**)

Further analysis of double-strand DNA breaks revealed that the PD model induced an increase in damage, evidenced by elevated levels of phosphorylated histone gamma H2AX in both cell lines. In contrast, therapeutic treatments resulted in a reduction of DNA damage, regardless of the treatment type (Fig. 5A, B). Interestingly, detailed cell cycle profiling showed no significant changes except in the co-treatment group for HT-22 neurons (Fig. 5C–F). Specifically, co-treatment led to G0–G1 phase reduction and G2/M extension compared to the PD model. Additionally, the cell cycle-related proteins p21 and p27 were upregulated in the PD model (with the exception of p27 for SH-SY5Y). A significant increase in p27 was noted in the HT-22 line under combined treatment compared to the PD model, while a decrease in p21 was observed in the SH-SY5Y line across all treatment groups. No other significant changes were detected (Fig. 5G–L). Finally, ATP levels in the HT-22 cell line increased in the PD model and combined treatment group, while a decrease was observed in both monotherapy groups (Fig. 5M). Completely opposite results were observed in the SH-SY5Y line, namely ATP levels decreased in the PD model and increased in the other treatment groups (Fig. 5N).

### Neurite Outgrowth Potentiation Mediated by Nerve Growth Factor (NGF)

**Fig. 6:**
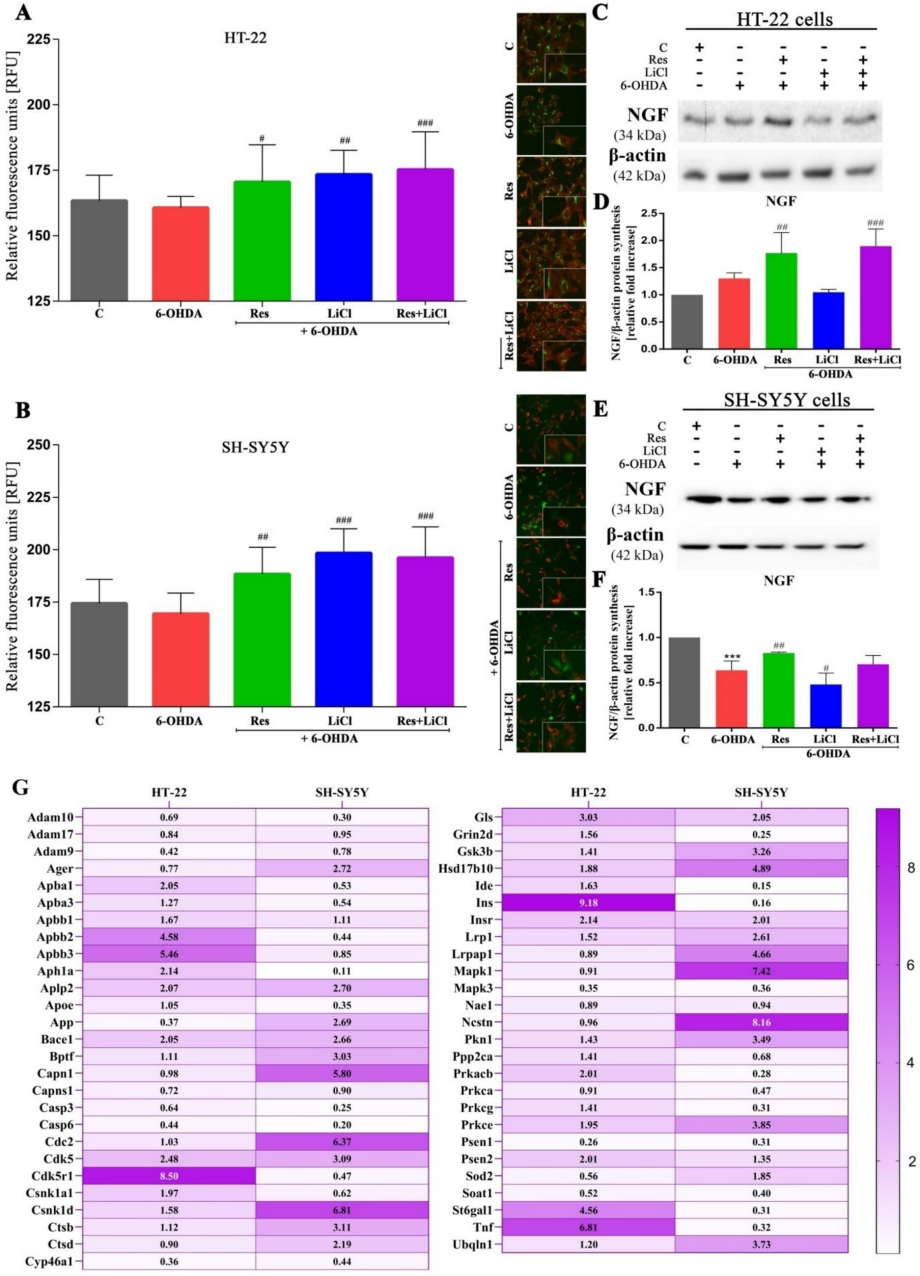
Stimulation of neurite outgrowth (**A**, **B**) by nerve growth factor activity (**D**, **F**) of HT-22 and SH-SY5Y cells treated with Res, LiCl and Res + LiCl. Representative immunoblots are presented (**C**, **E**). Heat map represents gene expression associated with neurodegenerative disease pathology, biochemistry and genetics for the HT-22 and SH-SY5Y lines treated with Res + LiCl in combination (**G**)

In the next stage, an increase in neurite outgrowth was observed across all therapeutic groups, including both monotherapies and combined treatments, with the exception of the 6-OHDA group. These results were consistent in both cell lines (Fig. 6A, B). Additionally, NGF expression was examined. Upregulation was observed with resveratrol and combined treatments in the HT-22 cell line compared to the control PD model (Fig. 6C, D). In contrast, the SH-SY5Y cell line exhibited downregulation of NGF in the PD model group, with further downregulation in the LiCl monotherapy group. Only the Res-treated group showed an increase in NGF expression (Fig. 6E, F).

### Neurodegenerative Genes Regulation Dynamics Involved in Therapies-Applied Response

Finally, a screening was conducted only for the combined treatment group, focusing on genes implicated in neurodegenerative diseases. In the HT-22 cell line, the highest increases in expression were observed for Apbb2, Apbb3, Cdk5r1, Ins, St6gal1 and Tnf genes, while the lowest expression increases were noted for Adam9, App, Casp6, Cyp46a1, Mapk3 and Psen1. In contrast, the combined treatment induced a different pattern of gene activation in the SH-SY5Y cell line. The highest upregulation was observed for Capn1, Cdc2, Csnk1d, Hsd17b10, Lrpap1, Mapk1 and Ncstn genes, while the lowest expression levels were observed for Adam10, Aph1a, Apoe, Casp3, Casp6, Grin2d, Mapk3, Prkacb, Prkeg, Psen1, St6gal1, or Tnf (Fig. 6G).

## Discussion

One of the primary challenges in the field of neuroscience is the lack of effective treatment methods for neurodegenerative diseases. PD is among these disorders and its treatment is currently focused solely on symptom elimination. Therefore, searching for effective and minimally invasive therapeutic methods is highly requested (Mathur et al. 2023).

In this study, we demonstrate that combined treatment with resveratrol and lithium chloride is more effective than monotherapy. This outcome appears to be mediated by a mechanism involving intersecting antioxidant and autophagic pathways via p62/Keap1/Nrf2 signaling. We provide evidence that the compounds used in combined therapy, due to their different mechanisms of action, could be developed into an effective PD therapy and, because they include natural components, may also be used as a preventive measure in the elderly. Our studies used both healthy and cancerous neuronal cells and our results were cell line-specific. The use of two cell lines with different physiological properties provided valuable insights into the underlying mechanisms of various responses.

First, we analyzed metabolic activity and reactive oxygen species levels to select individual doses for further study. For the toxic 6-OHDA substance, we selected a dose that increased ROS levels and decreased metabolic activity to induce a PD model based on oxidative stress (Kim et al. 2024). Conversely, we chose resveratrol and lithium chloride doses that did not increase ROS levels and caused a slight increase in metabolic activity.

Then, we conducted a metabolic activity assay to verify the PD model and evaluate the therapeutic effects of the compounds tested. In healthy HT-22 cells, increased activity was observed for treatments with Res, LiCl, or their combination. Conversely, the positive control with the PD model showed a decrease in metabolic activity. Interestingly, in cancer cells, the observed activities were reversed, indicating that the chosen compounds effectively targeted PD. These results suggest that cancer cell metabolism significantly differs from healthy cells and likely requires more energy for growth and division. This finding is consistent with previous studies showing increased metabolic activity in SH-SY5Y cells treated with a neurotoxin that induces PD (Kurnik-Łucka et al. 2022).

Next, we conducted a series of tests to demonstrate the antioxidant properties of the compounds, particularly the combined treatment, which exhibited high antioxidant activity compared to the PD model. Overall, the therapy reduced ROS/RNS levels and increased total antioxidant capacity. Furthermore, the compounds increased reduced glutathione levels, a defense mechanism against free radicals. These observations indicate that the substances have strong antioxidant effects, particularly evident in the combined therapy. For the PD model, an increase in ROS/RNS and a decrease in GSH were observed, likely indicating the activation of a non-enzymatic cellular defense mechanism against free radicals. Additionally, increased oxidative stress is one of the initial cellular responses to environmental stress and is linked to neurodegenerative diseases (Barnham et al. 2004; Liu et al. 2017). Notably, the results were consistent across both cell lines. Previous studies have shown that resveratrol can reduce ROS levels by regulating autophagy via the AMPK-mTOR pathway (Song et al. 2018) and lithium chloride has been shown to improve cognitive impairments by inhibiting apoptosis and oxidative stress through the GSK-3β/β-catenin signaling pathway (Wang et al. 2020). Downregulation of the NF-κB protein in the PD model, involved in cellular responses to free radicals and antioxidant responses, supports our findings (Lingappan 2018). Our results indicate disrupted antioxidant processes and redox imbalance due to excessive free radical formation. The activation of this protein in therapeutic systems may suggest its role in maintaining homeostasis and combating oxidative stress. Data confirm that lithium regulates apoptosis and expression of apoptotic genes dependent on NF-κB and MAP kinase (Németh et al. 2002). Other studies show that oxidative stress generated by 6-OHDA activates NF-κB and the expression of apoptotic proteins such as Bcl-2 may depend directly on NF-κB (Blum et al. 2001).

We further examined changes in the activity of proteins involved in the p62/Keap1/Nrf2/ARE pathway in response to applied therapies. In HT-22 cells, there was a decrease in ARE activity in the PD model and an increase in the system treated with lithium chloride only. Notably, we did not observe activation of Nrf2 pathway proteins, indicating that the Nrf2 pathway associated with autophagy was not activated in the mouse hippocampal neuron PD model. The lithium chloride treatment effectively increased ARE activity, indicating a potential enhancement of the antioxidant response. However, the lack of activation of Nrf2 pathway proteins suggests that this increase might not be due to the canonical Nrf2 pathway, but rather through alternative mechanisms or pathways (Lau et al. 2010). In SH-SY5Y cells, different results were observed. Increased ARE activity was noted in the PD model and a decrease in the system with resveratrol. The p62/Nrf2/Keap1/ARE pathway was also activated. The PD model itself increased ARE activity, suggesting an initial compensatory response to oxidative stress. The resveratrol treatment, however, reduced ARE activity, which may indicate that resveratrol either reduces oxidative stress or downregulates the pathway (Zhang et al. 2019a). The observed activation of the p62/Nrf2/Keap1/ARE pathway supports the hypothesis that resveratrol affects this pathway differently in SH-SY5Y cells compared to HT-22 cells. Overall, the greatest activation of proteins in the p62/Keap1/Nrf2 pathway was observed in the combined set, indicating its strong antioxidant effect. It appears that NRF2 induces the p62 protein, which in turn increases Nrf2 activity and, along with reduced Keap1 levels, may stimulate autophagy, consistent with the activation of the LC3A/B protein. Our results are consistent with other studies showing that 6-OHDA activates the p62/Keap1/Nrf2 pathway, protecting SH-SY5Y cells from ferroptosis (Sun et al. 2020). Additionally, resveratrol inhibits ROS production by activating the Nrf2 pathway and regulating NF-κB protein (Zhang et al. 2019b). Moreover, analyzing the expression of key proteins involved in autophagy, we observed a lack of activation of this pathway in the HT-22 PD model, consistent with the absence of p62/Keap1/Nrf2 pathway activation. Notably, the autophagy-inhibiting complex was activated in this system, along with proteins involved in apoptosis. The absence of autophagy activation and the concurrent activation of autophagy-inhibiting complexes and apoptosis-related proteins suggest a cellular environment favoring cell death rather than survival. This is consistent with the lack of Nrf2 pathway activation, indicating that the cells are unable to mount an effective antioxidant response, leading to the promotion of apoptosis over autophagy (Towers et al. 2019; Occhiuto et al. 2023). Further results were consistent with previous findings where p62/Keap1/Nrf2 pathway activation was observed in the SH-SY5Y PD model and proteins involved in the autophagy pathway were activated. The results reaffirm the cell line-specific responses and this specificity underscores the importance of considering cell type in the evaluation of therapeutic interventions for neurodegenerative diseases. The autophagy process can serve as a cell survival pathway or an apoptosis-inhibiting pathway. However, it is often correlated with apoptosis or can occur as an alternative mechanism (Eisenberg-Lerner et al. 2009).

Particular attention was given to the co-treatment system. In this system, we observed the highest overall increase in proteins associated with autophagy and high activation of apoptotic proteins. Additionally, the highest activity of the p62/Keap1/Nrf2 pathway was also noted. The activation of both the p62/Keap1/Nrf2 pathway and the autophagy pathway suggests a cellular response aimed at mitigating oxidative stress and promoting cell survival. This indicates a protective mechanism where autophagy is upregulated to clear damaged proteins and organelles, which is in line with the activation of the Nrf2 pathway known for its role in cellular defense against oxidative stress (Ma 2013). mTOR-dependent autophagy through oxidative stress and apoptosis may be important therapeutic targets for neuroprotection. Data confirm that 6-OHDA leads to autophagy activation via AMPK/mTOR (Arsikin et al. 2012), while resveratrol also affects the autophagic pathway to alleviate damage in a PD model (Shen et al. 2023). Regarding combined therapy, studies by Lee et al. (2023) also indicate the beneficial properties of this approach. Authors demonstrated that combination therapy using metformin and OSMI-1 results in a correlation between autophagy and apoptosis mechanisms, potentially causing a much stronger effect than monotherapy (Lee et al. 2023).

As expected, we observed an increase in DNA damage in the PD model and a decrease in the treated sets. The greatest elimination of damage was observed in the combined group, likely due to the high activation of the previously mentioned pathways. Others suggest that resveratrol protects cells from damage in a rat PD model (Khan et al. 2010), while 6-OHDA causes significant DNA damage (Bernstein et al. 2011). Interestingly, cell cycle analysis showed no major changes except for the combined set in HT-22 cells, where a decrease in G0–G1 phase cells and an increase in G2/M phase cells compared to the PD control were noted. The observed decrease in the G0–G1 and the increase in the G2/M phase suggest that the combination treatment accelerates the transition of cells through the G1 phase, pushing more cells into the G2/M phase where cell division occurs. This could indicate enhanced cell proliferation or a response to stress, leading to an accumulation of cells preparing for mitosis. This may also suggest the activation of adaptive mechanisms to the treatment, aiming to balance cell survival and proliferation in the context of neurodegeneration (Stark and Taylor 2004). These results were correlated with the activation of p21 and p27 proteins, which are crucial in modulating the cell cycle, especially in the transition from the G1 to the S phase. The link between the shortened G0–G1 phase and the activation of these proteins suggests that the combination treatment enhances the regulatory control exerted by p21 and p27, facilitating a quicker transition through the G1 phase. By decreasing cellular damage, the Nrf2 pathway might enable cells to bypass the G1 checkpoint more efficiently, leading to increased proliferation and survival (Liu et al. 2021).

Further, in the HT-22 PD model, we observed an increase in ATP levels, which could be associated with the activation of apoptosis, that is considered an energy-intensive process and studies indicate the maintenance of high ATP levels in cells during the process of cell death (Zheng et al. 1991; Zamaraeva et al. 2005). In the SH-SY5Y PD control model, we noted a decrease in ATP levels, which may be related to excessive free radical formation and consequently reduced ATP production. Moreover, ATP could have been utilized for the high metabolic activity observed in cell growth and proliferation in this system. Other authors have also observed a decrease in ATP levels following 6-OHDA treatment, associated with high oxidative stress (Tirmenstein et al. 2005; Simões et al. 2022). For the co-treatment in both cell lines, we observed high ATP levels, likely explained by the activation of Nrf2, as this protein influences ATP synthesis (Dinkova-Kostova and Abramov 2015). By promoting ATP synthesis, the co-treatment likely contributes to overall mitochondrial health, which is vital for maintaining cellular homeostasis and preventing apoptosis. We also observed stimulation of neurite outgrowth, likely in response to NGF activation (Liu et al. 2007). Other studies confirm that resveratrol can activate NGF-related signaling pathways, such as the PI3K/Akt pathway, leading to neurite elongation and improved neuronal function. Neurite growth is crucial in the context of neurodegenerative diseases like Alzheimer's and Parkinson's, where neuron degeneration is a major issue. Stimulation of neurite growth by resveratrol may contribute to the regeneration and repair of damaged neurons, offering potential therapeutic benefits (Tang et al. 2017).

In the final stage, we screened the combined treatment system, focusing on genes involved in neurodegenerative diseases. We observed an increase in the expression of Apbb2 and Apbb3. These genes are associated with the transport and processing of the beta-amyloid precursor protein and are involved in the regulation of cell death processes (Sobue et al. 2023). Proper expression of Cdk5r1 is crucial for neuron differentiation, synapse formation and axon regeneration (Ao et al. 2022). The increased expression of this gene observed in mouse hippocampal cells may be reflected in the neurite outgrowth assay, where we demonstrated high growth for the combined system. Studies by Fei et al. confirm that JIP1 phosphorylation via Cdk5 promotes neuroaxonal outgrowth in mice (Fei et al. 2024). Additionally, the upregulation of the Ins gene activated by the co-treatment may be associated with a protective function in the HT-22 cell line. Ins deficiencies lead to neurodegenerative and depressive states (Pomytkin et al. 2018). Others have also shown increased insulin gene expression following resveratrol treatment in mouse αTC9 cells (Xie et al. 2013). We also demonstrated the activation of genes regulating inflammatory states such as ST6Gal1 and Tnf. These genes are mediators in neuroinflammation associated with neurodegeneration (Amin et al. 2022; Makarava et al. 2022). Studies by Ndebele et al. show that Tnf mediates the induction of apoptosis (Ndebele et al. 2008). These results are reflected in our studies, where we also observe the activation of apoptotic proteins as a result of the co-treatment.

In the SH-SY5Y cell line model treated with resveratrol and lithium chloride, we observed high expression of Capn1 associated with increased synaptic plasticity and neuroprotection (Su et al. 2020). In the 6-OHDA-induced Parkinson’s disease mouse model, Capn1 activation improves motor and cognitive functions (Guo et al. 2022). Moreover, the upregulation of Cdc2 and Csnk1d is related to cell cycle regulation, apoptosis and cell differentiation (Joseph et al. 2020; Wang et al. 2023). The expression of these genes likely translates to proper cell cycle regulation and apoptosis processes in SH-SY5Y cells in the combined treatment system. Additionally, high expression of genes such as Hsd17b10, Lrpap1, Mapk1 and Ncstn is responsible for regulating mitochondrial proteins and synaptic functions related to neuron survival. Each of these genes participates in neuroprotection through various mechanisms (Lee et al. 2014; Singh et al. 2014; Ahmed et al. 2020). Overall, in the HT-22 line, there was activation of key genes in the cellular response to therapy, affecting processes such as cell signaling, inflammation and insulin metabolism, whereas in SH-SY5Y cells, genes involved in processes such as proteolysis, cell cycle, protein kinase signaling and lipid metabolism were engaged. The HT-22 and SH-SY5Y lines differ in gene activation patterns, suggesting that the molecular mechanisms involved in the response to combined therapy may be cell type-specific. Genes with the highest expression may play a key role in improving cellular functions and could be targets for further research on neurodegenerative therapies. Genes with the lowest expression may indicate protective or compensatory mechanisms that cells utilize in response to therapy. These results may have clinical significance, as understanding specific gene responses to therapies can aid in personalizing treatment and improving clinical outcomes for patients.

Despite the promising findings presented in this study, several limitations should be noted. The results were obtained using in vitro cell models which, although valuable for investigating fundamental mechanisms, cannot fully recapitulate the complexity of living organisms. Critical factors such as bioavailability, pharmacokinetics and interactions with diverse brain cell types require further investigation in in vivo models. Additionally, considering the chronic and progressive nature of Parkinson’s disease, long-term studies are necessary to evaluate the sustained efficacy and safety of the proposed therapy. Moreover, the pathogenesis of Parkinson’s disease is multifactorial and our study primarily focuses on oxidative stress and autophagy pathways. Future research should include additional mechanisms, such as neuroinflammation, to provide a more comprehensive understanding of the therapeutic potential.

## Conclusion

This study investigates the combined effects of resveratrol and lithium chloride in treating Parkinson’s disease. The results demonstrate that this combination therapy is more effective than individual treatments in reducing oxidative stress and enhancing antioxidant and autophagic pathways via the p62/Keap1/Nrf2 signaling mechanism (Aqeel et al. 2025). The combined therapy showed significant benefits, including reduced ROS/RNS levels, increased total antioxidant capacity and stimulated neurite outgrowth, indicating potential for therapeutic and preventive applications. Additionally, cell line-specific responses were noted, with differential gene activation patterns highlighting the complex mechanisms involved in the treatment of neurodegenerative diseases.

**Fig. 7:**
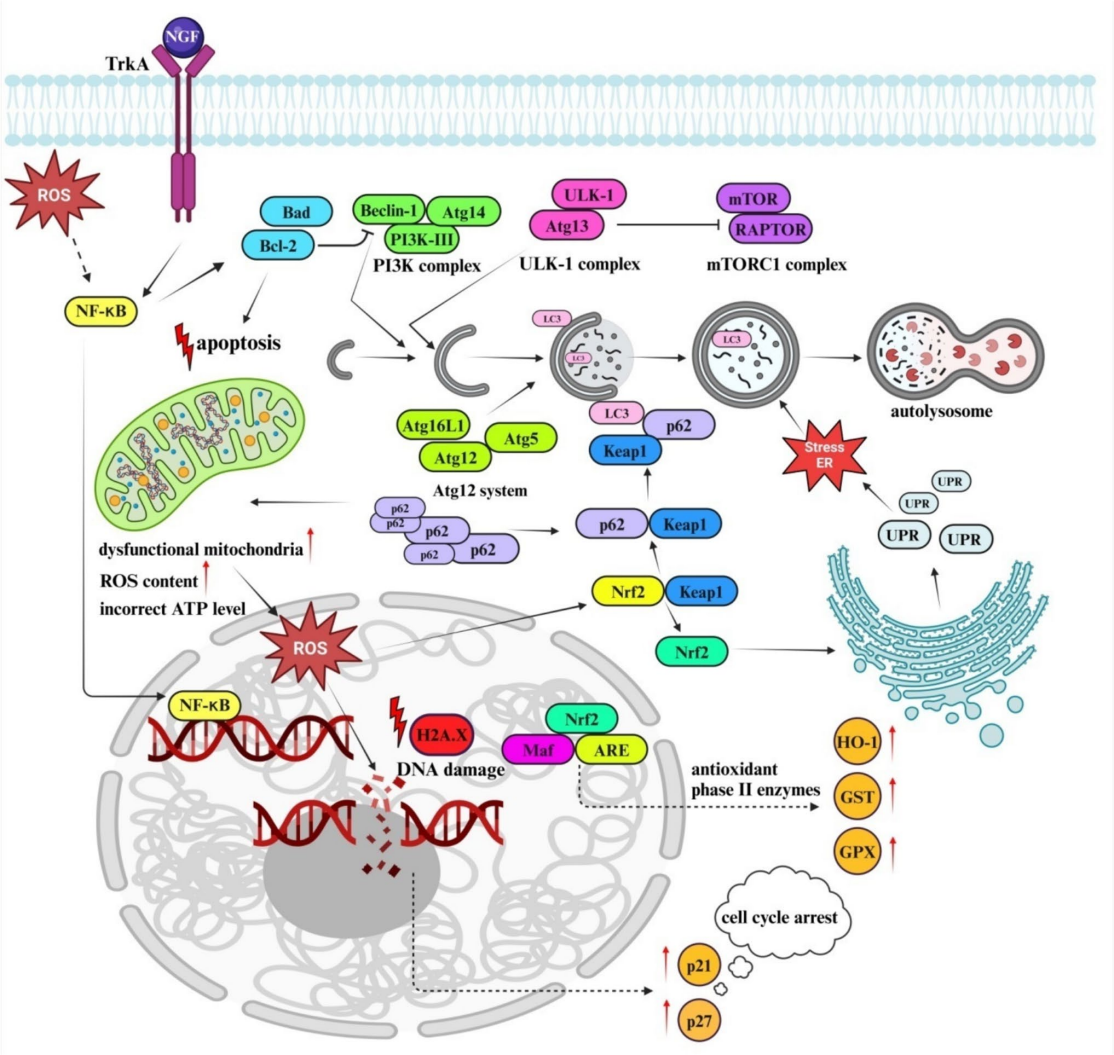
Proposed cell signaling mechanism controlled by resveratrol and lithium chloride. Created with BioRender

Expanding the scope of research to include other neurodegenerative diseases, such as Alzheimer’s, could provide insights into the broader applicability of combined resveratrol and lithium chloride therapy. Given the natural composition of the treatment, exploring its preventive potential in at-risk populations may offer significant public health benefits. This study lays a strong foundation for the development of innovative therapies for neurodegenerative diseases, emphasizing the importance of combined therapeutic approaches and the potential for natural compounds in clinical applications. Below, we present a proposal of the cell mechanism controlled by resveratrol and lithium chloride (Fig. 7).

## Supplementary Information

Below is the link to the electronic supplementary material.

Supplementary file1 (PDF 1860 KB)

## Data Availability

Data will be made available upon reasonable request.
