# Supplementary material for: Co-treatment Strategy Supports Neuroprotection by Intersecting p62-Keap1-NRF2 and Autophagy Signaling Pathways in the Cellular Model of Parkinson's Disease
Source: Cell Mol Neurobiol. 2025 Oct 16;45:85. doi: 10.1007/s10571-025-01610-9 (PMC12532526; doi:10.1007/s10571-025-01610-9)

<u>HT-22 cell line:</u>

β-actin | **ATG5**

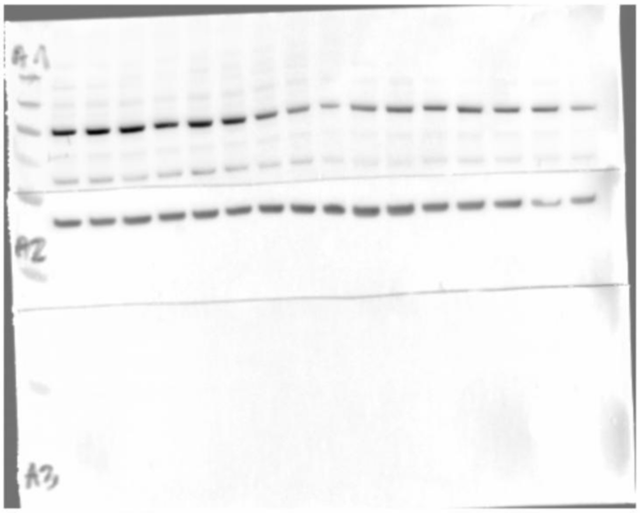

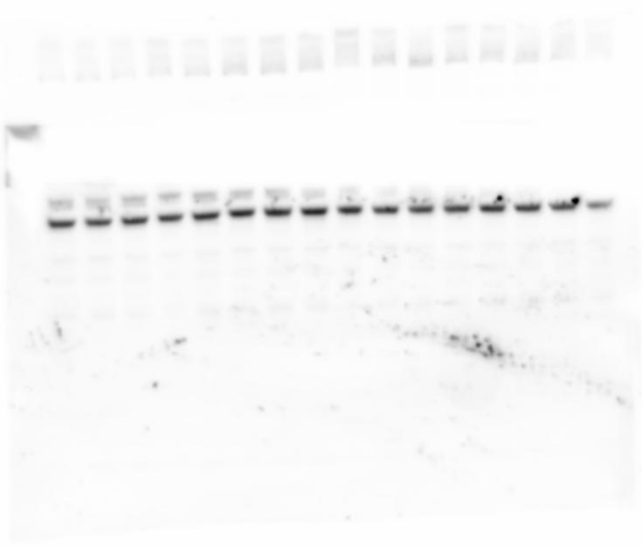

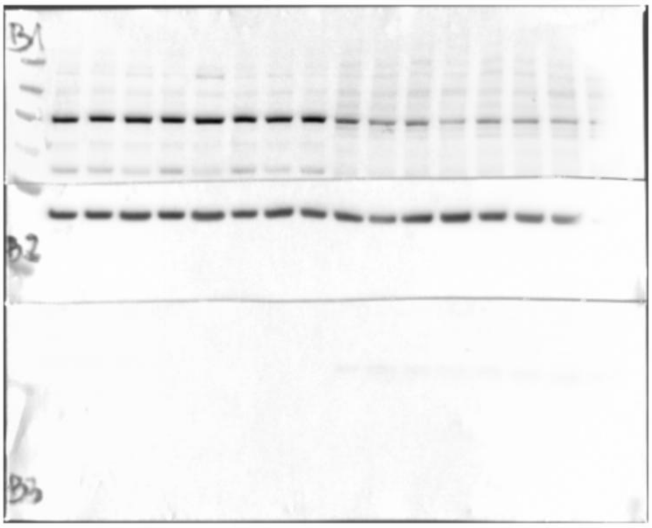

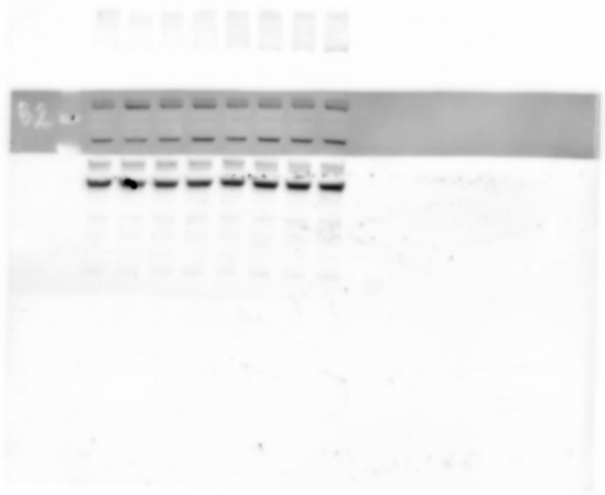

ATG13

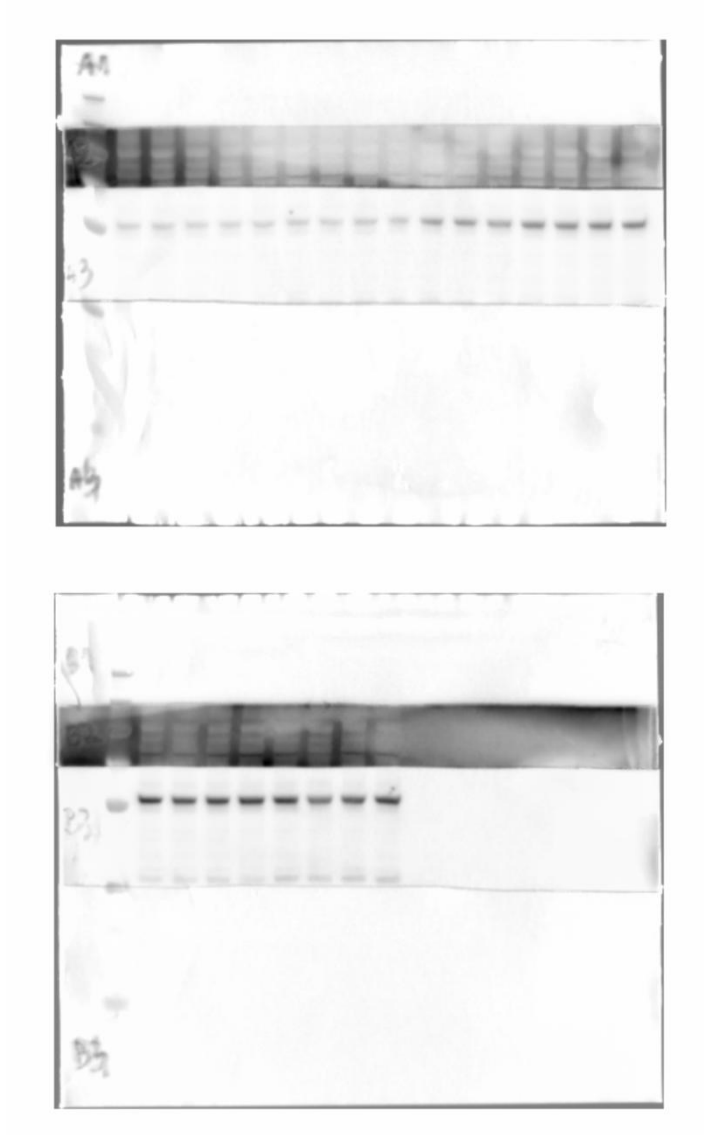

ATG14

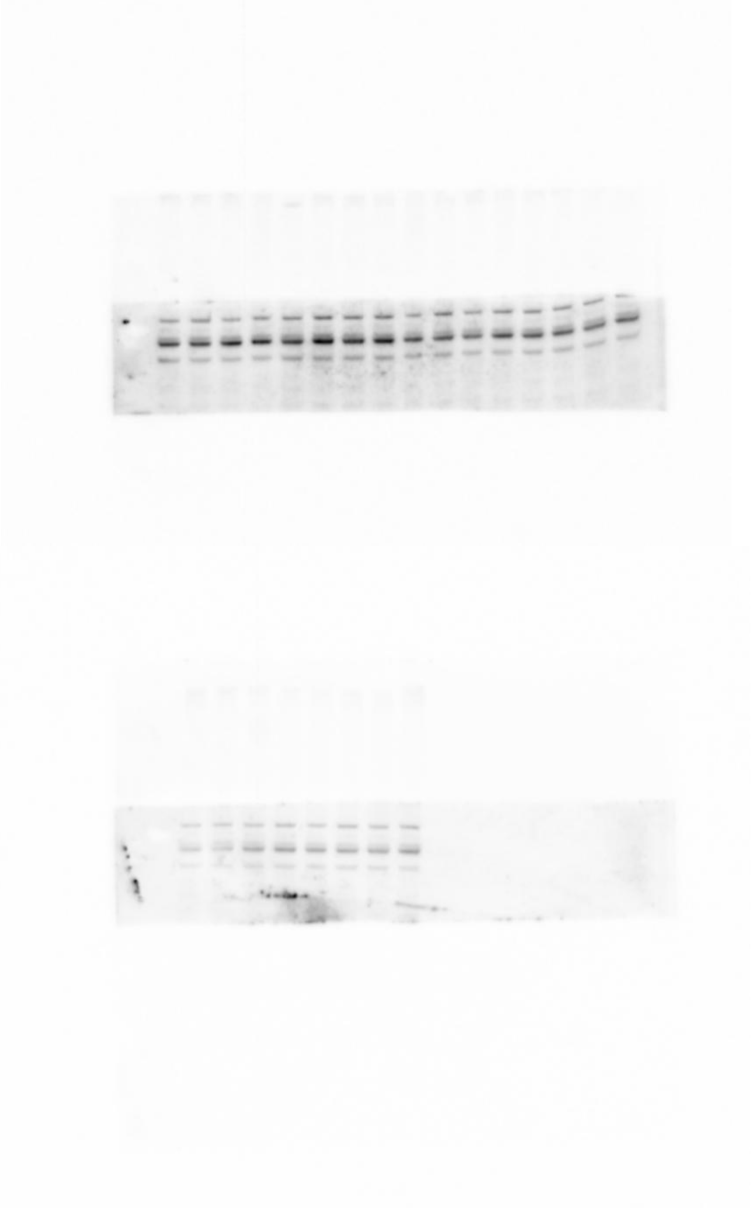

ATG16L1

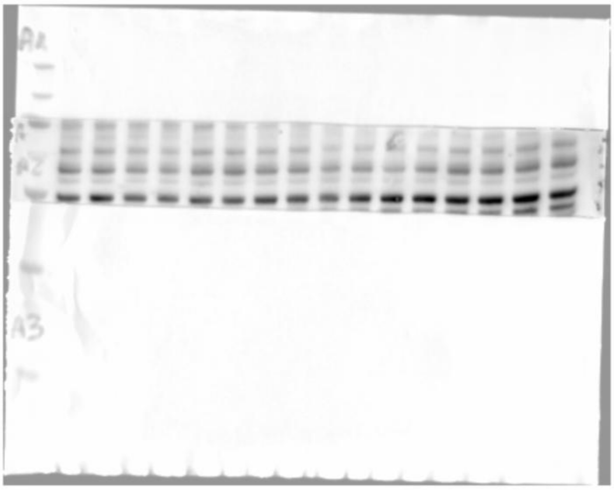

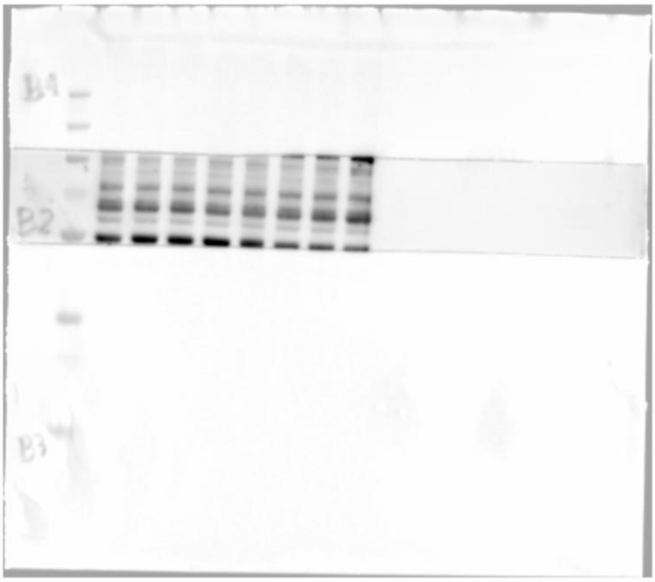

Bad

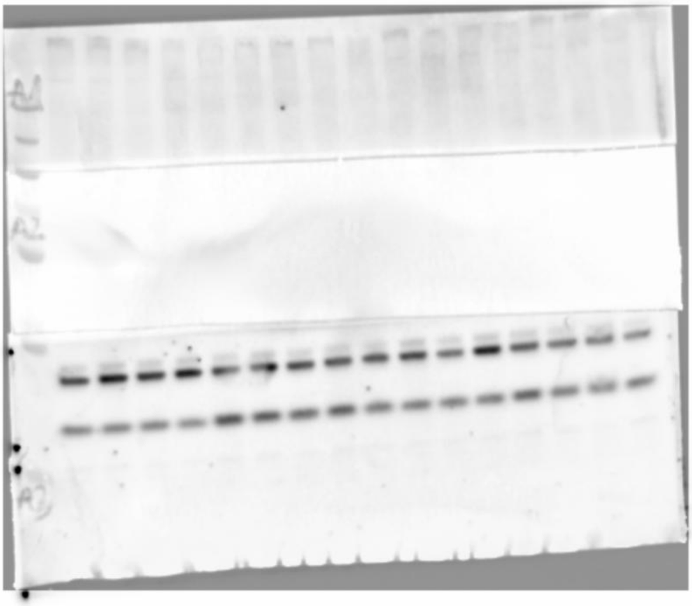

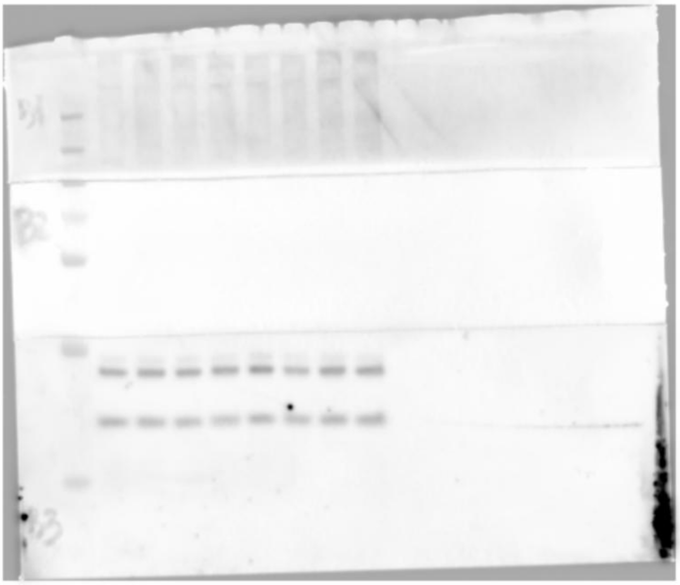

Bcl-2

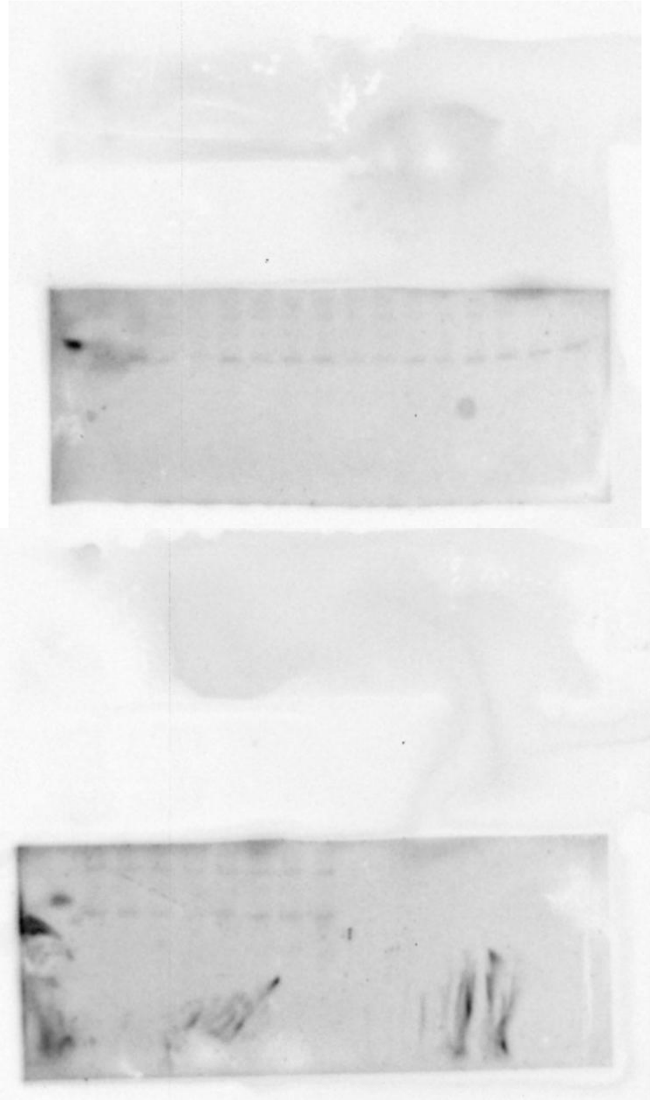

BECN1

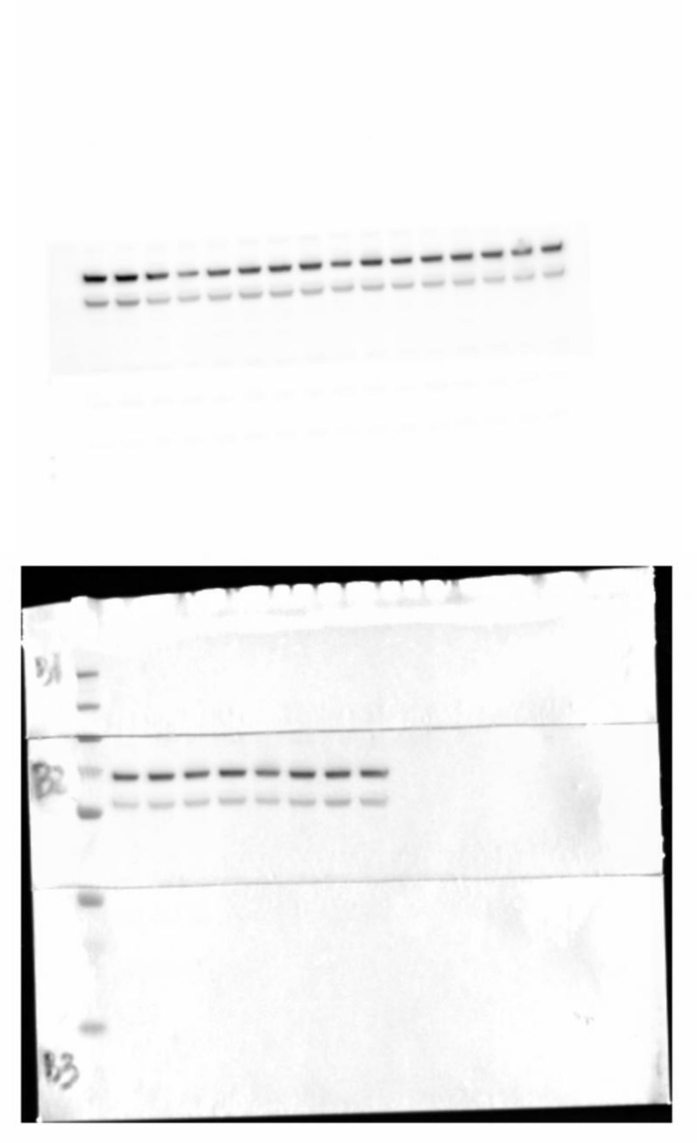

HO-1 | **Keap1**

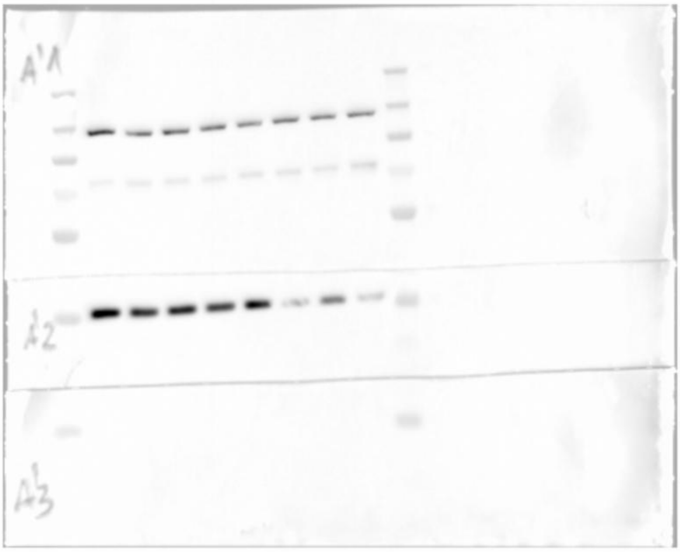

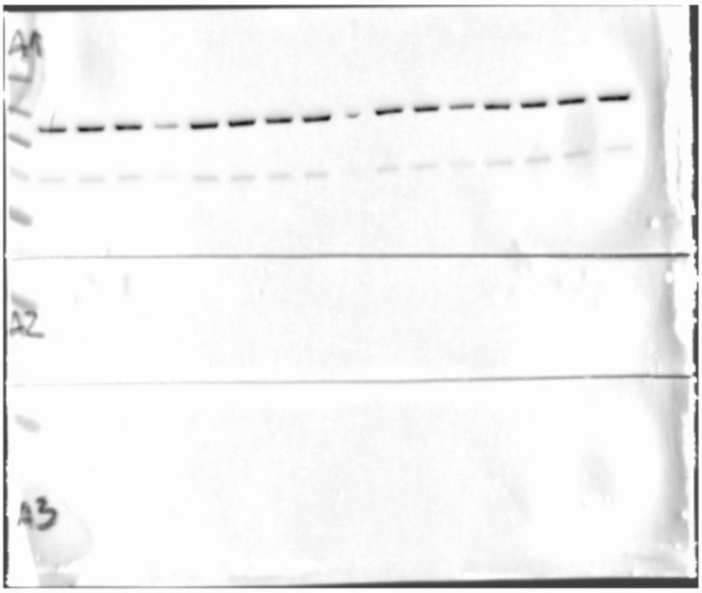

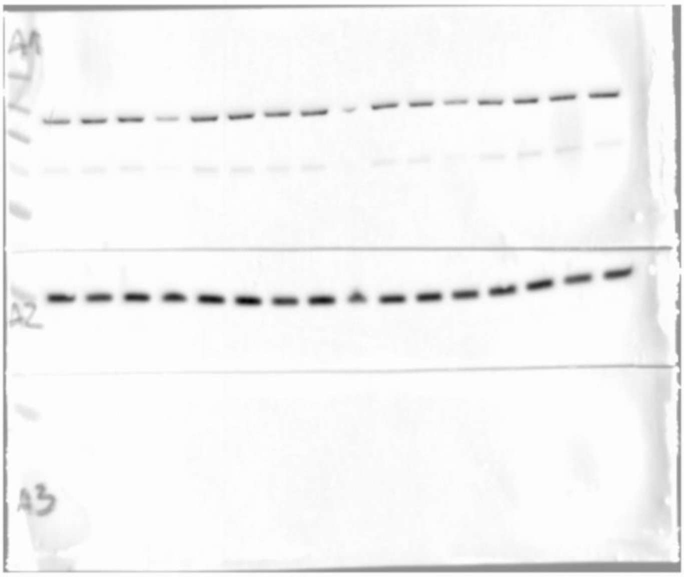

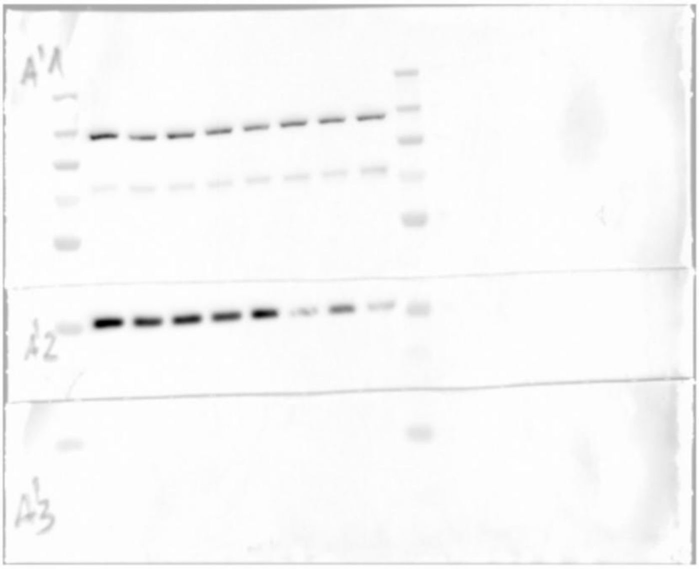

LC3A/B

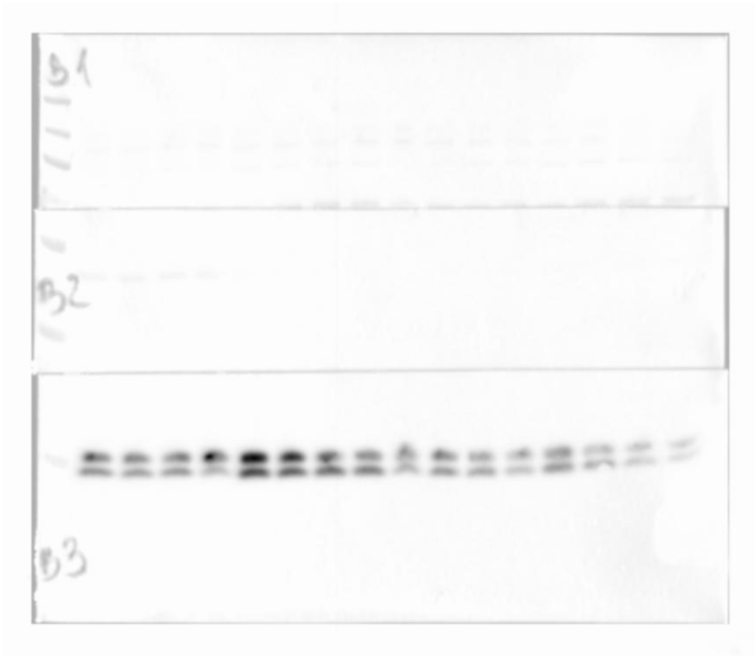

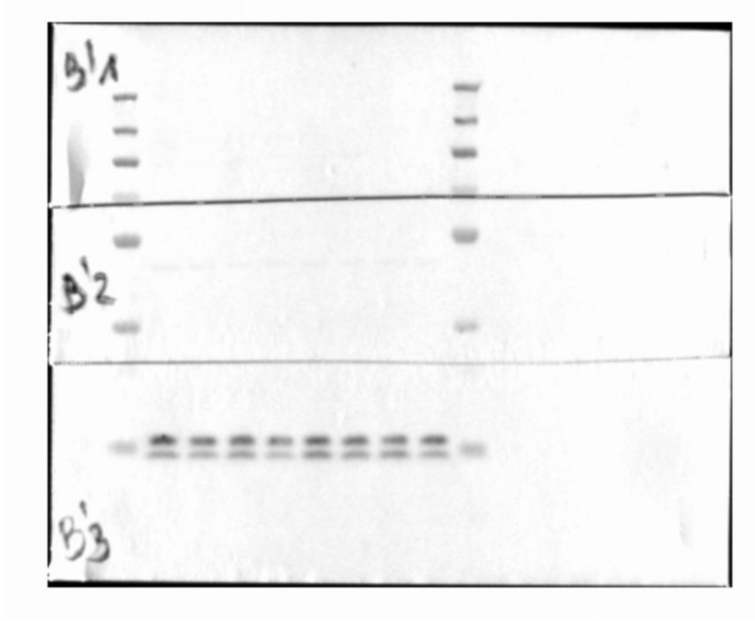

mTOR

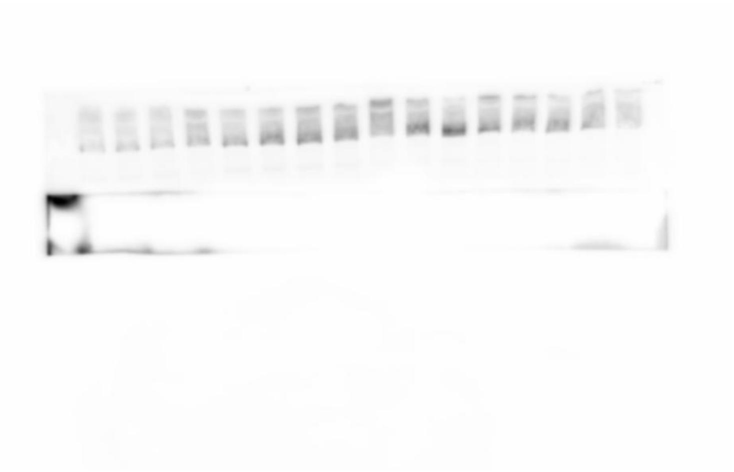

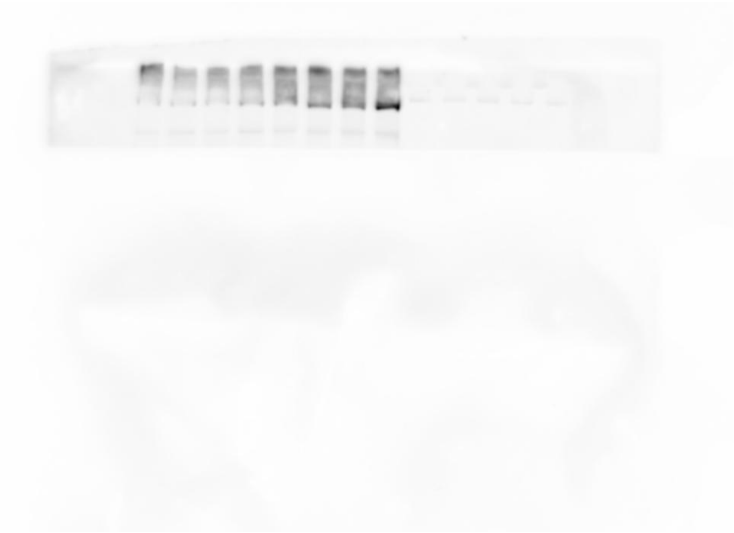

pSQTM

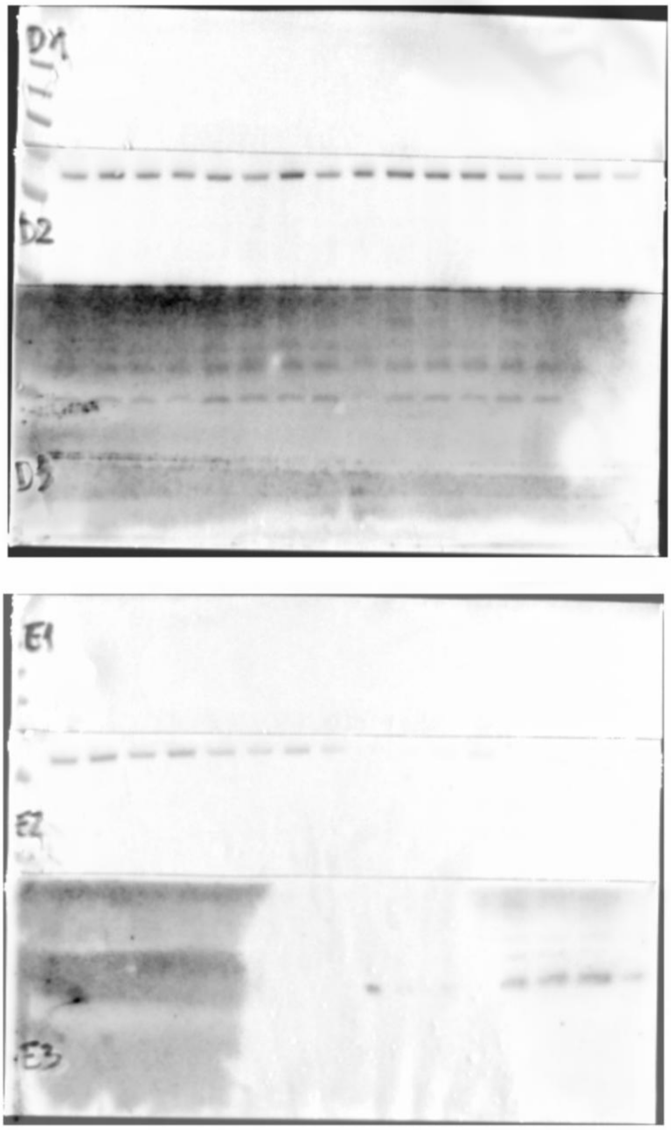

RAPTOR

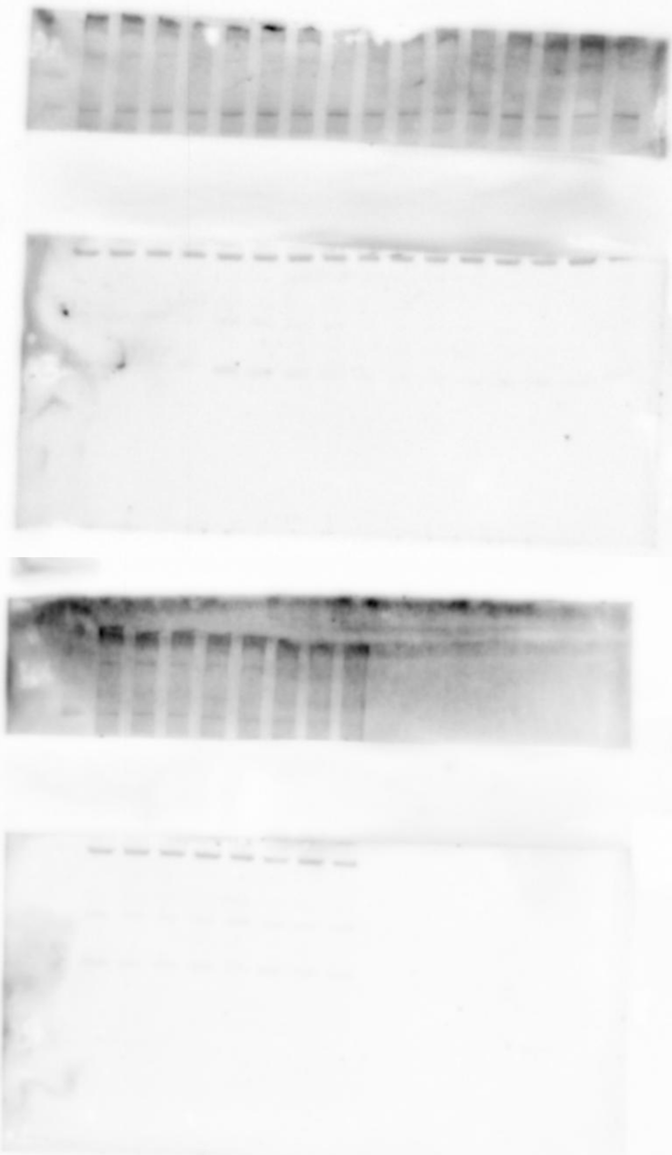

ULK-1

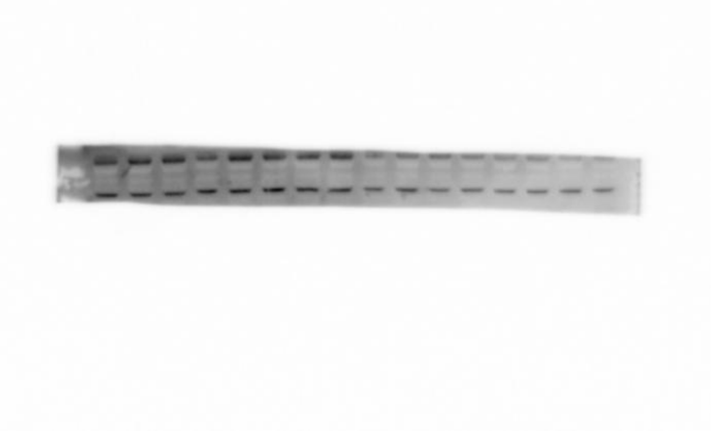

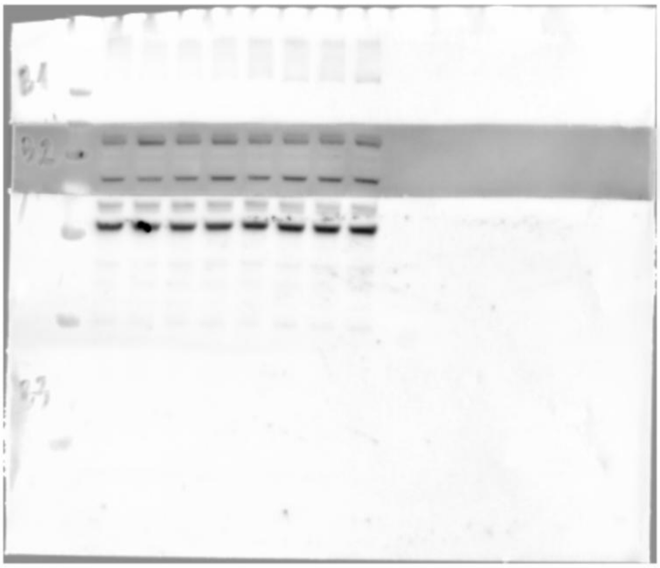

NF-κB

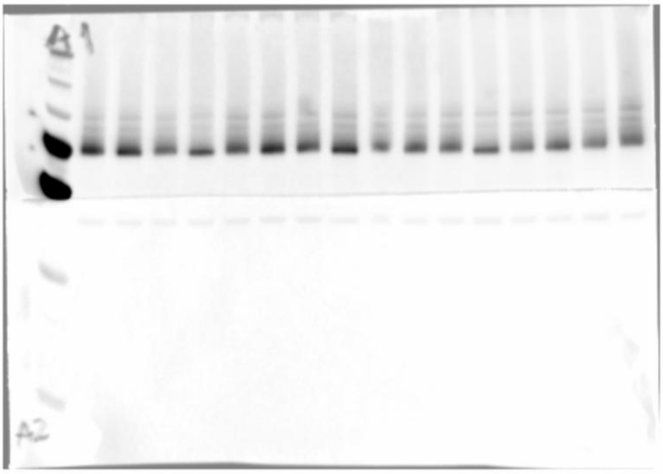

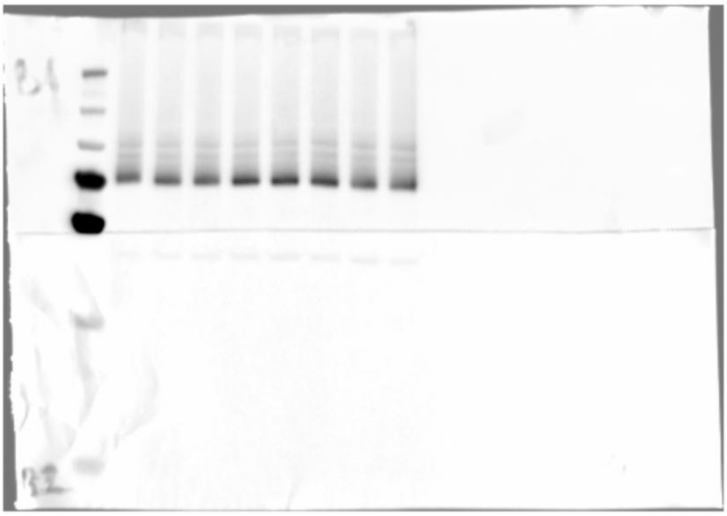

NGF

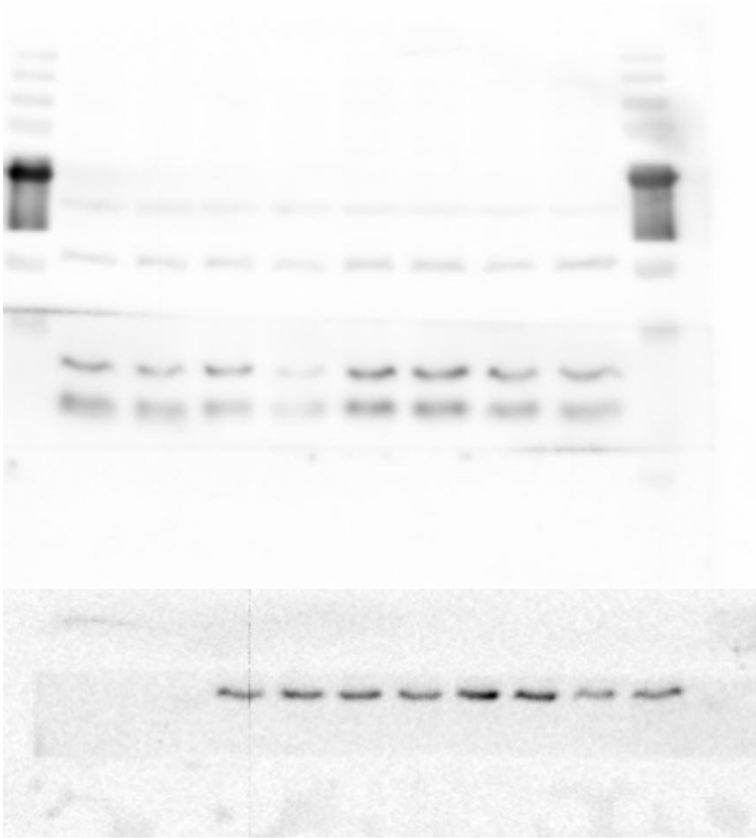

p21

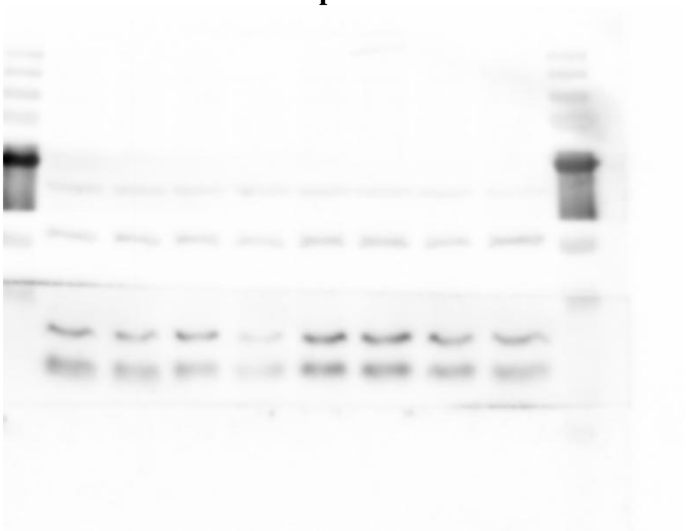

p27

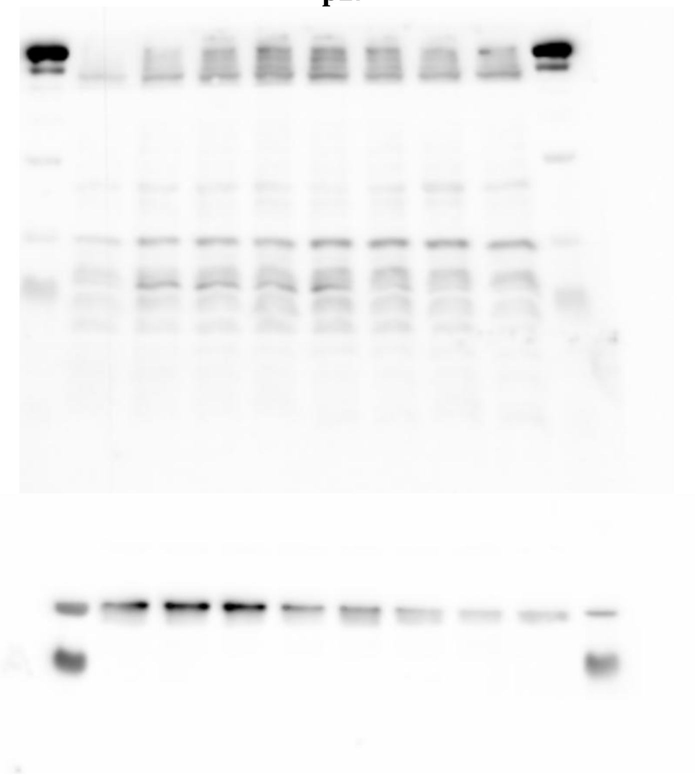

Nrf2

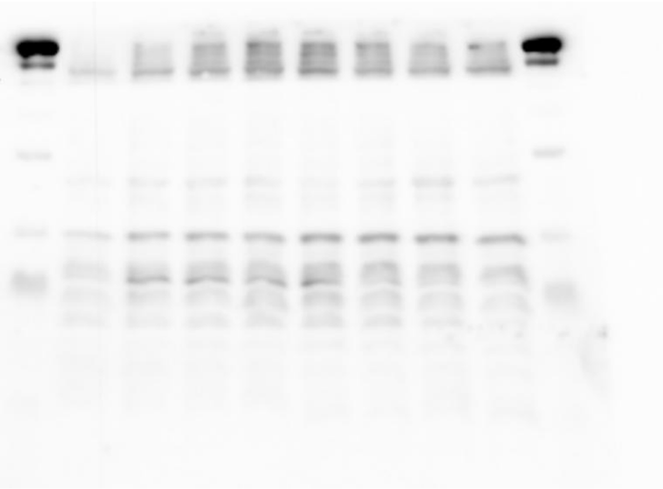

SH-SY5Y cell line:

β-actin

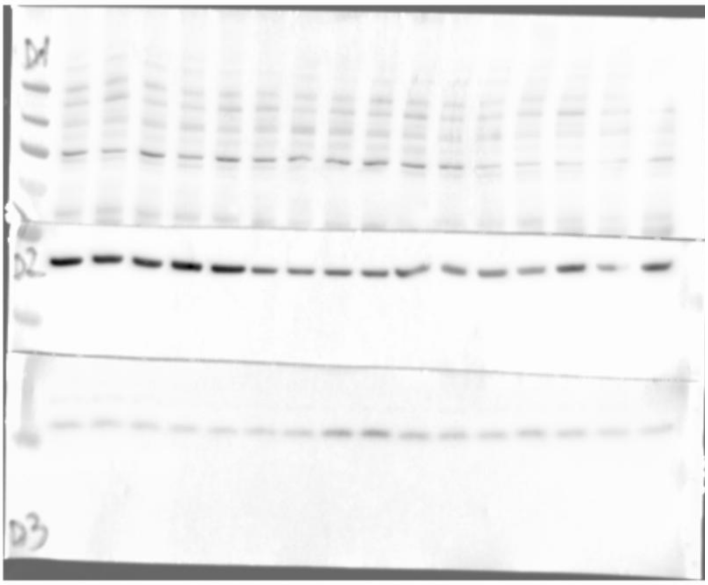

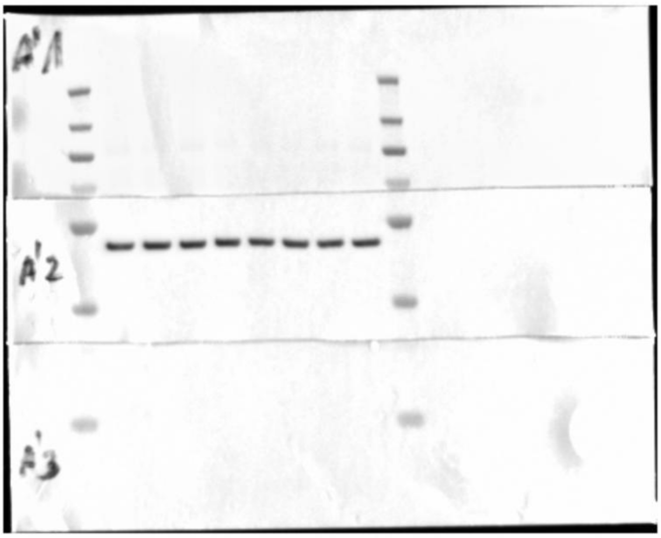

ATG5

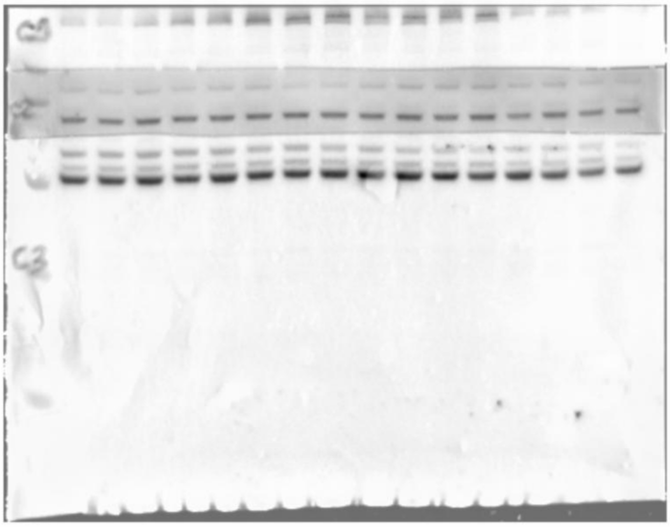

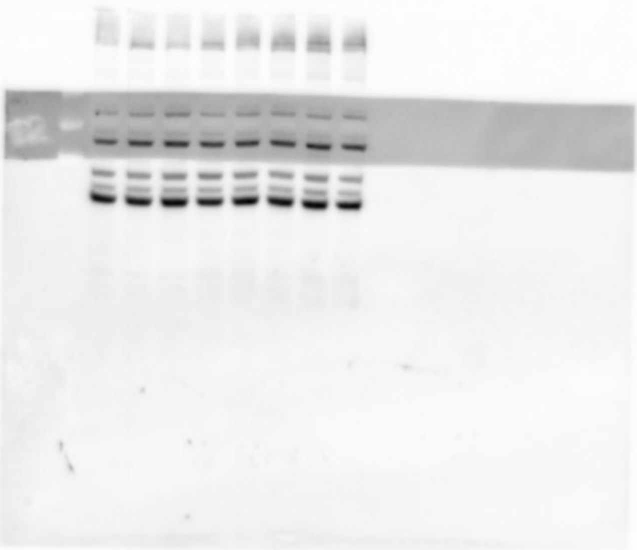

ATG13

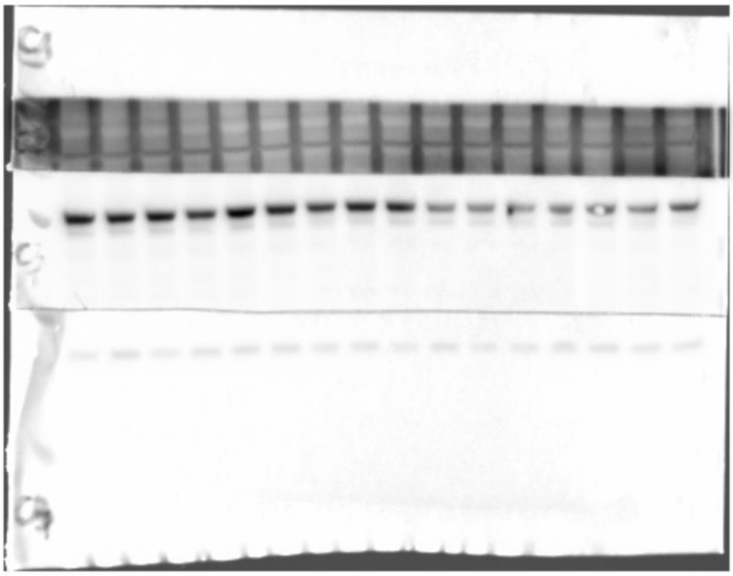

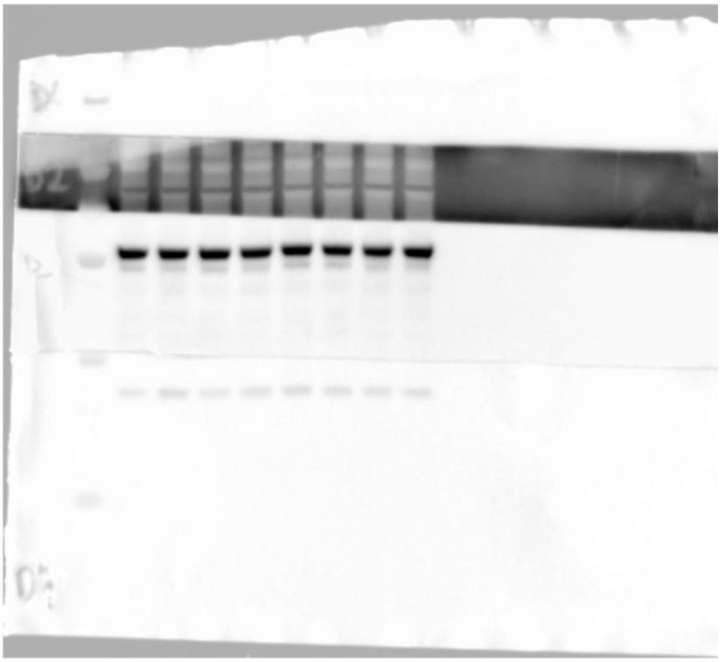

ATG14

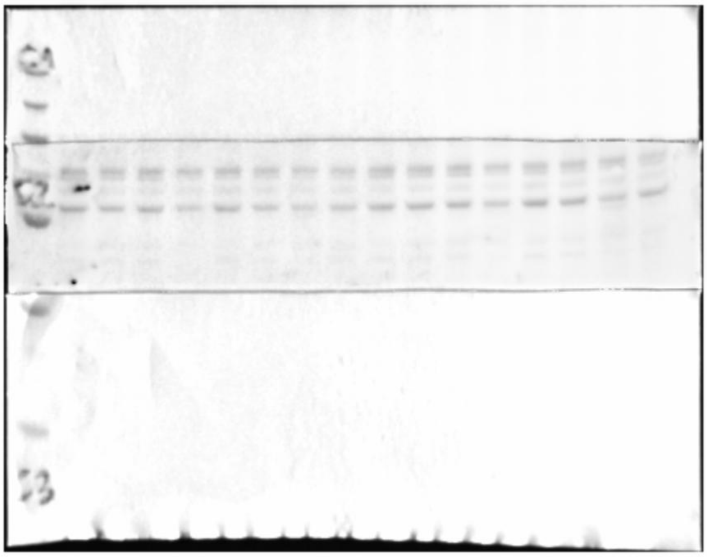

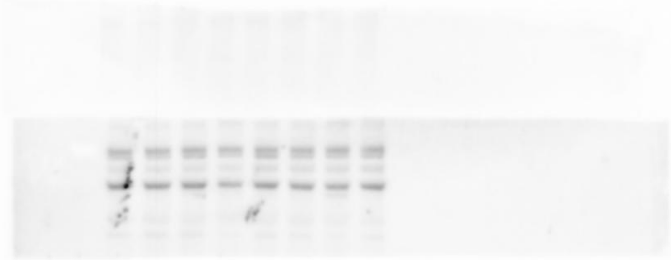

ATG16L1

BAD

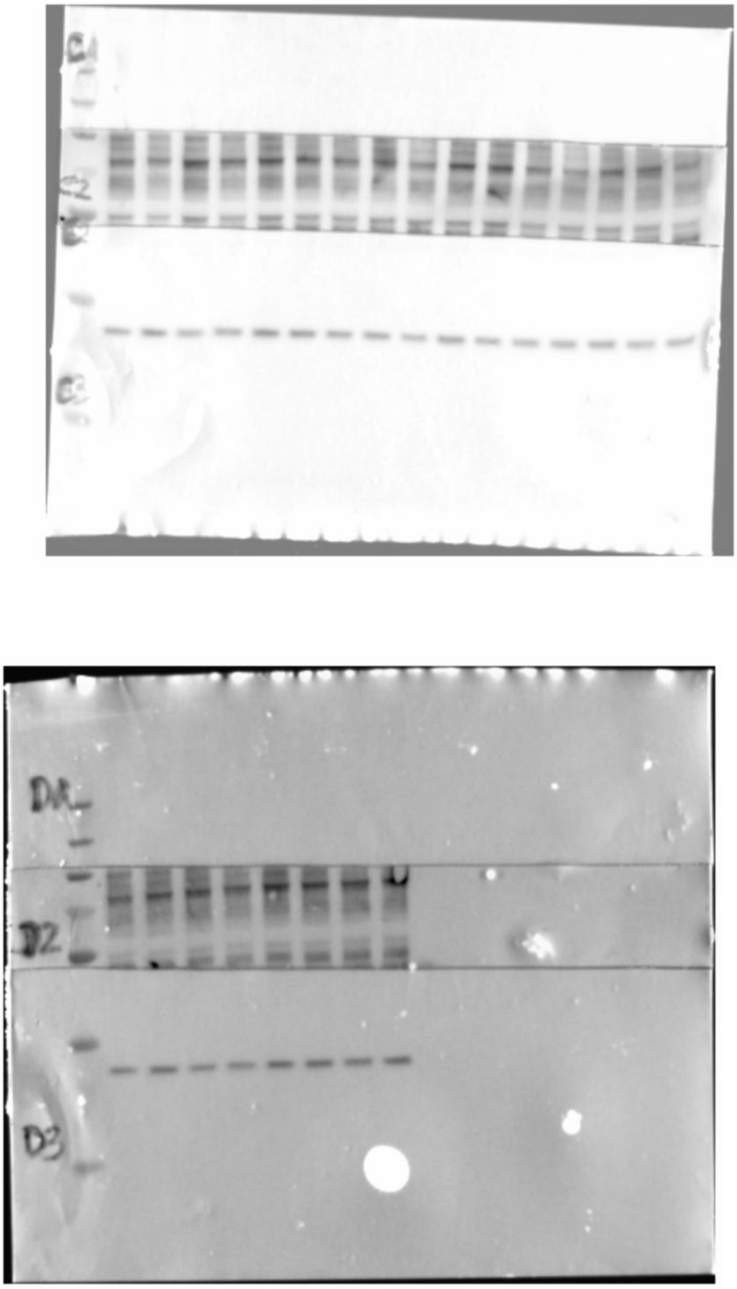

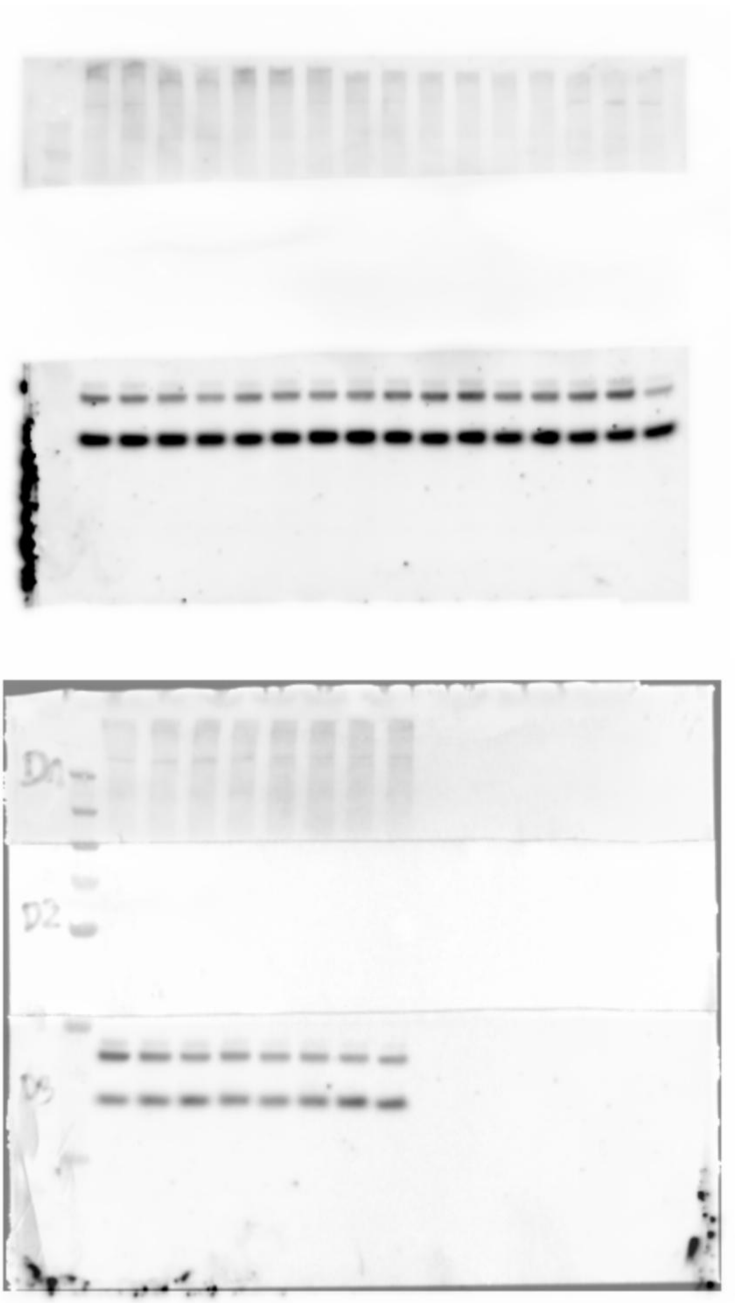

Bcl-2

BECN1

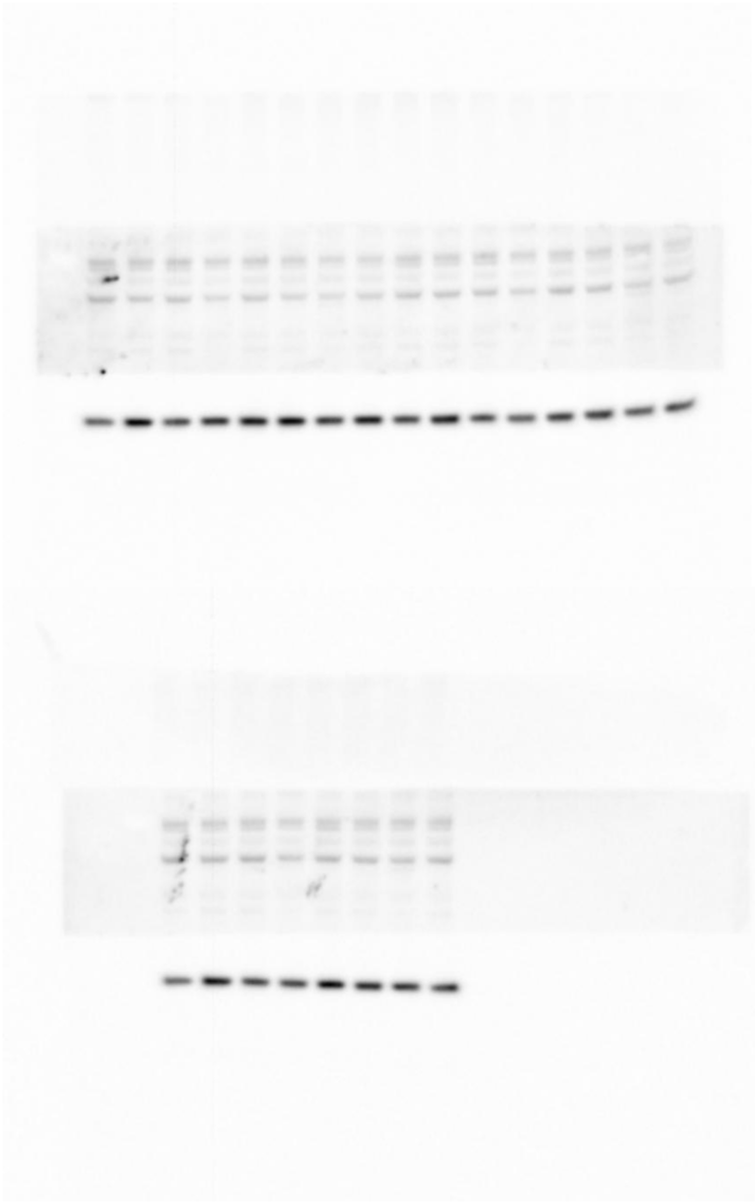

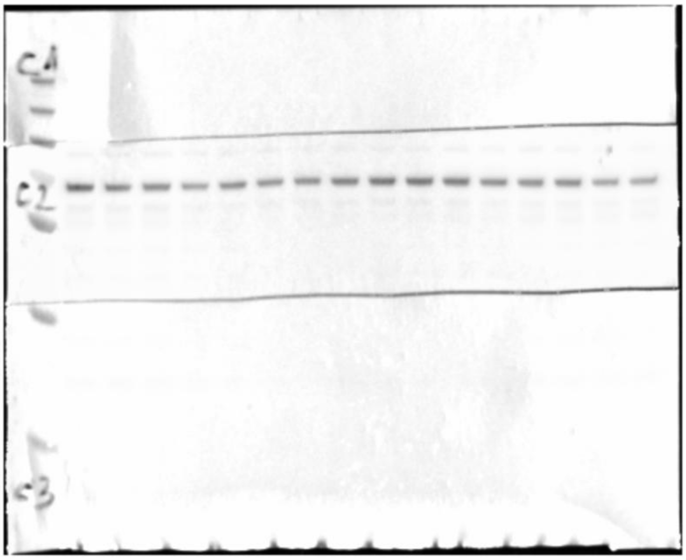

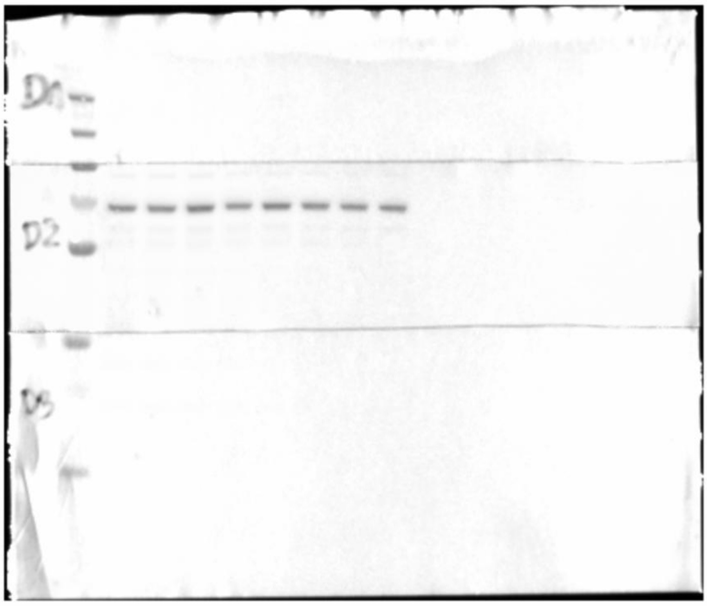

HO-1

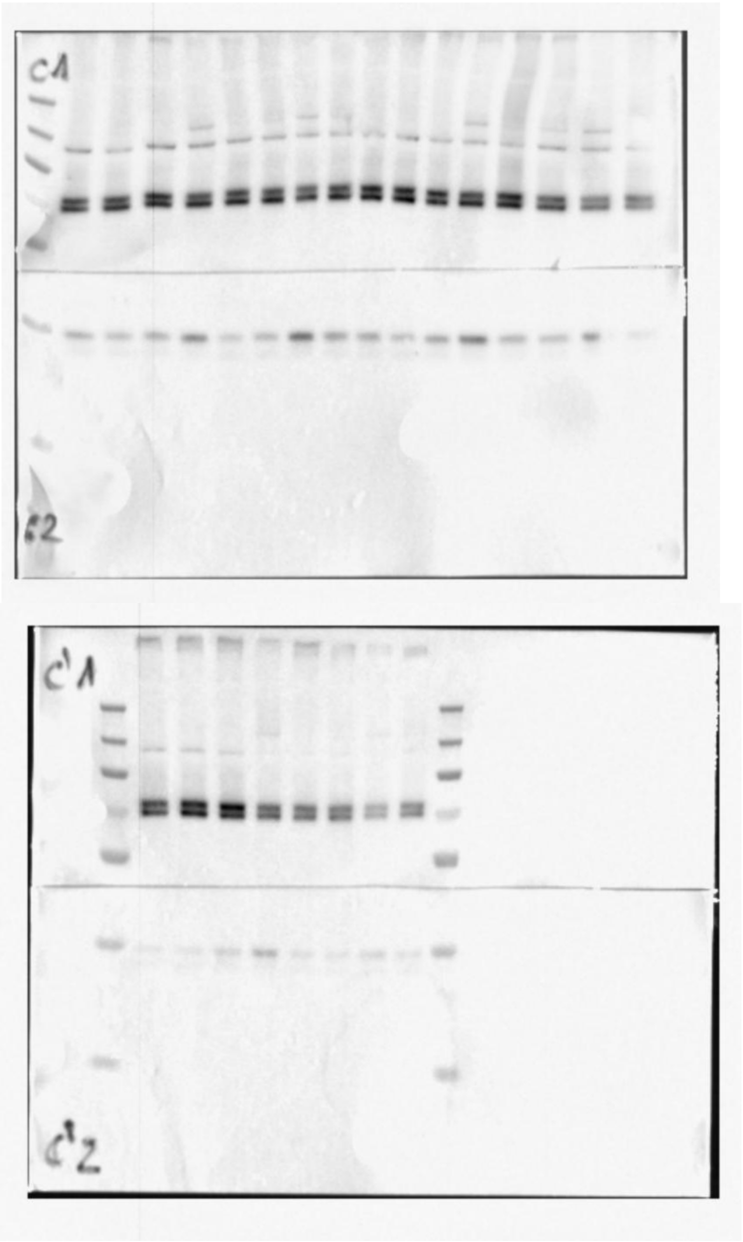

Keap-1

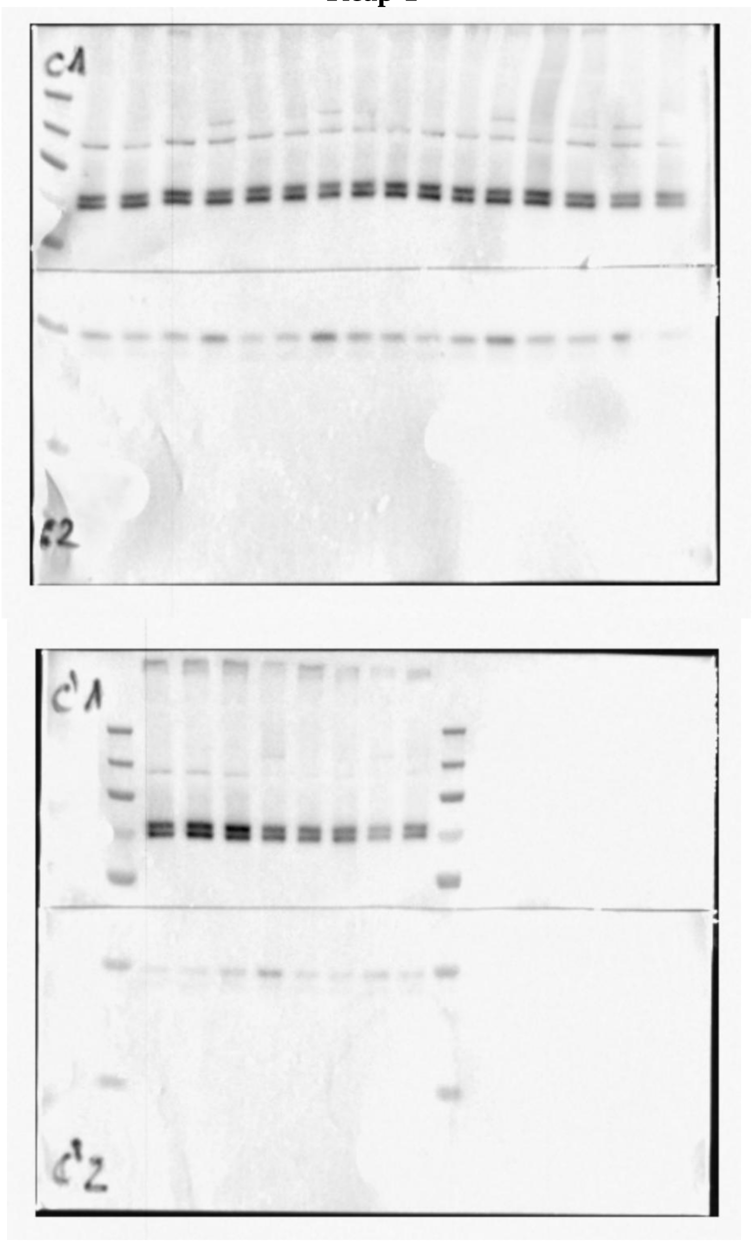

LC3A/B

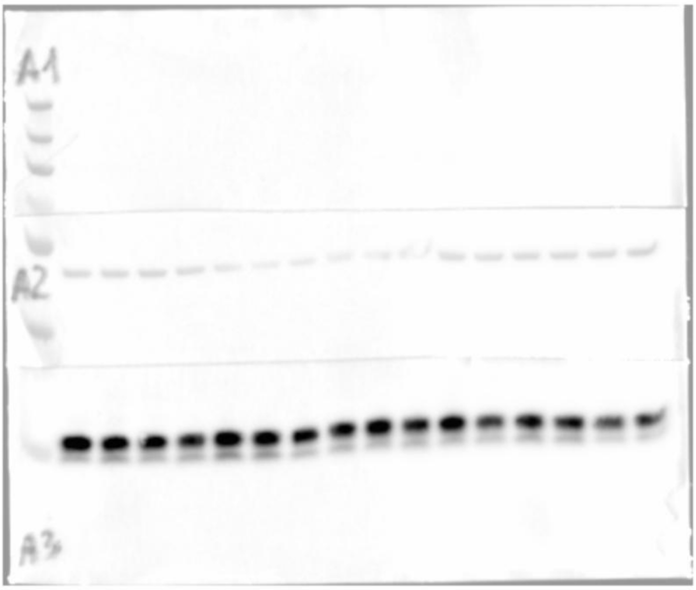

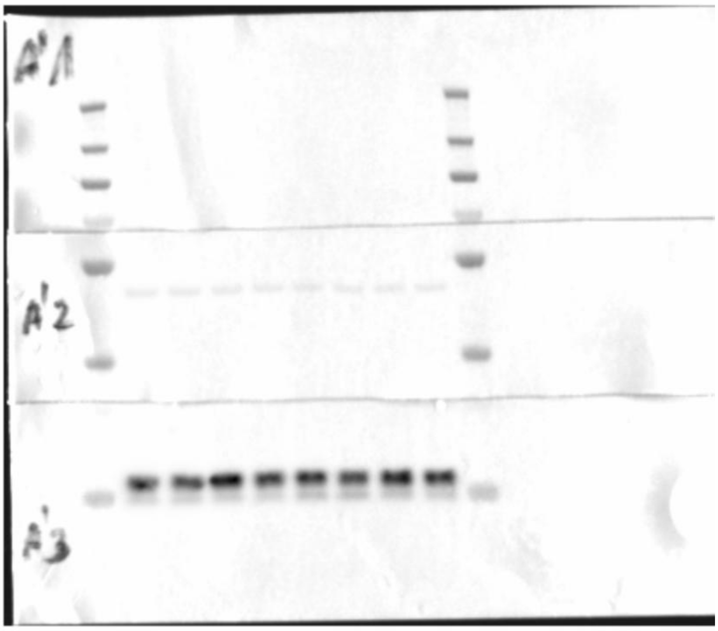

mTOR

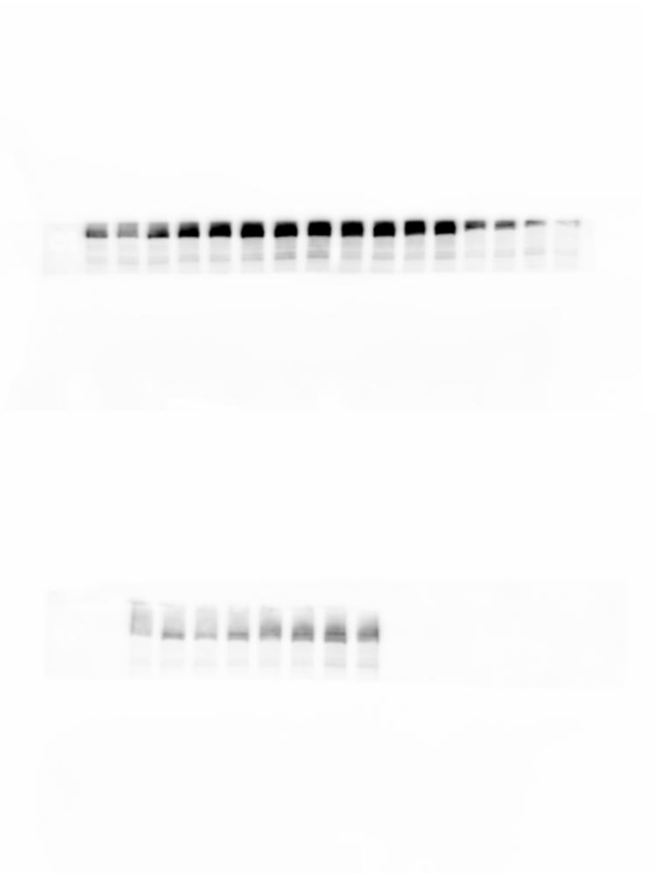

pSQTM

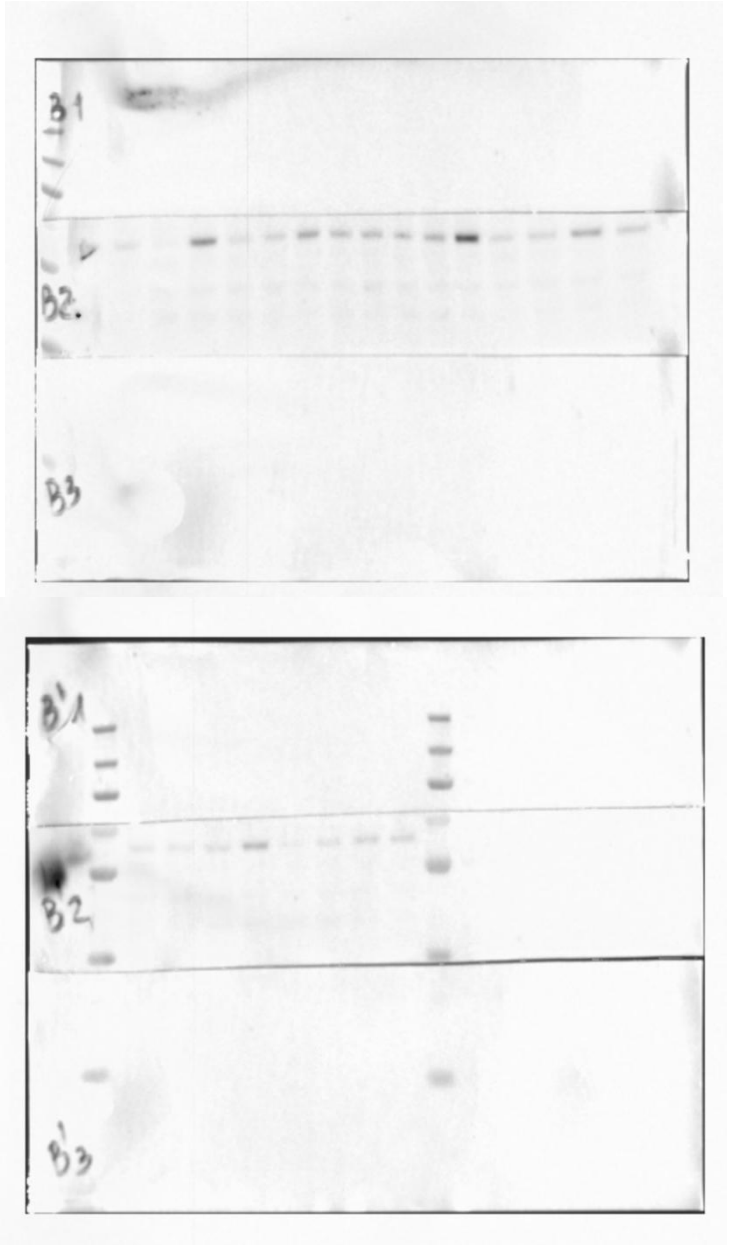

RAPTOR

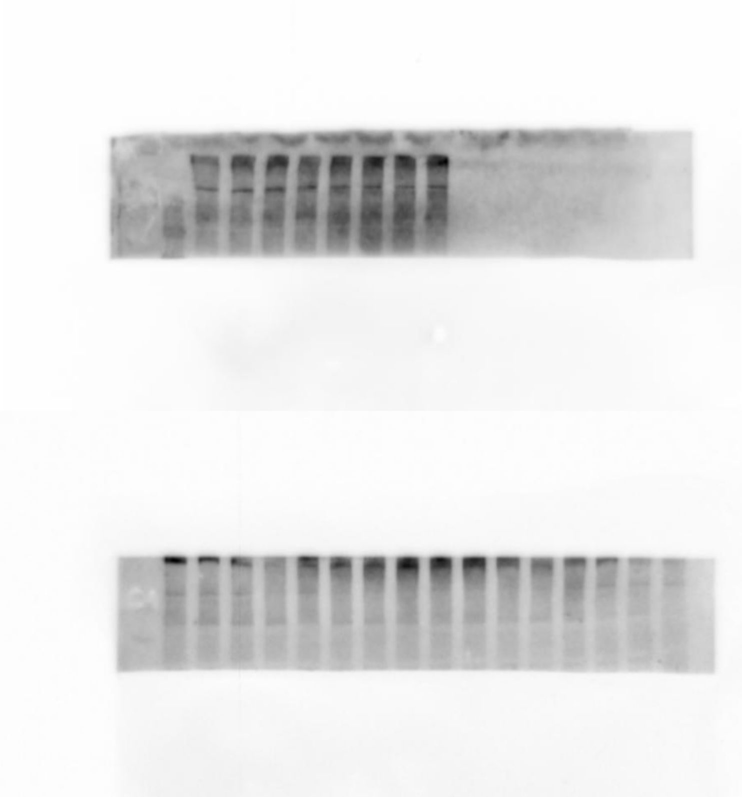

ULK1

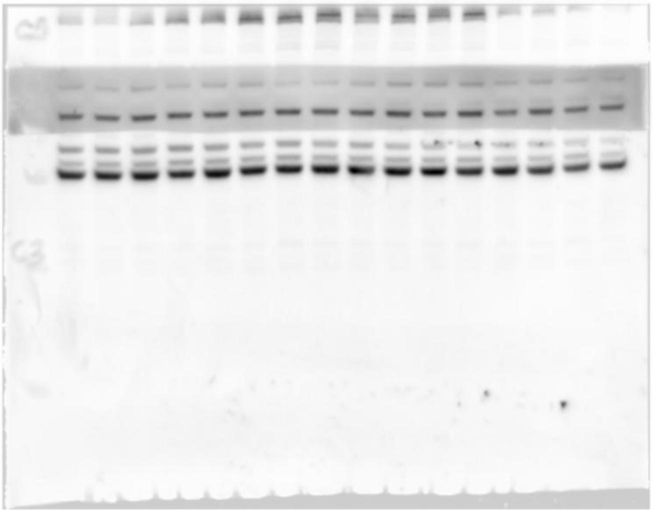

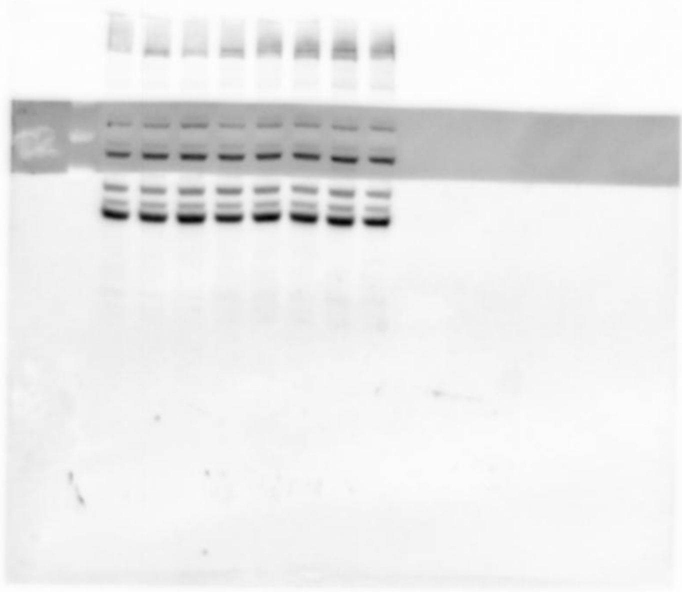

NF-κB

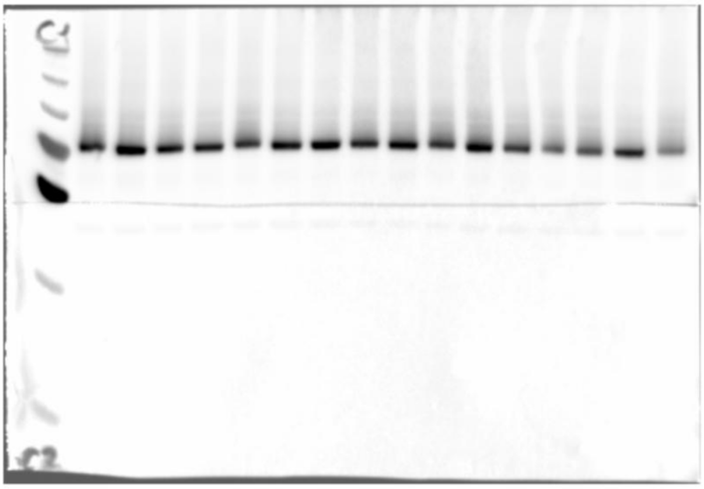

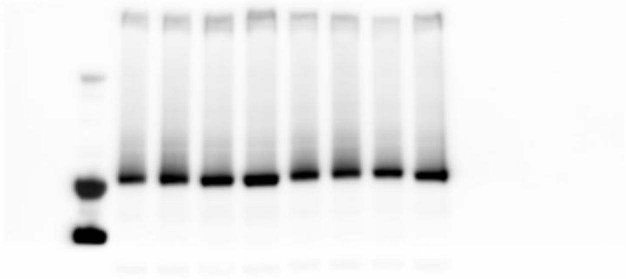

NGF

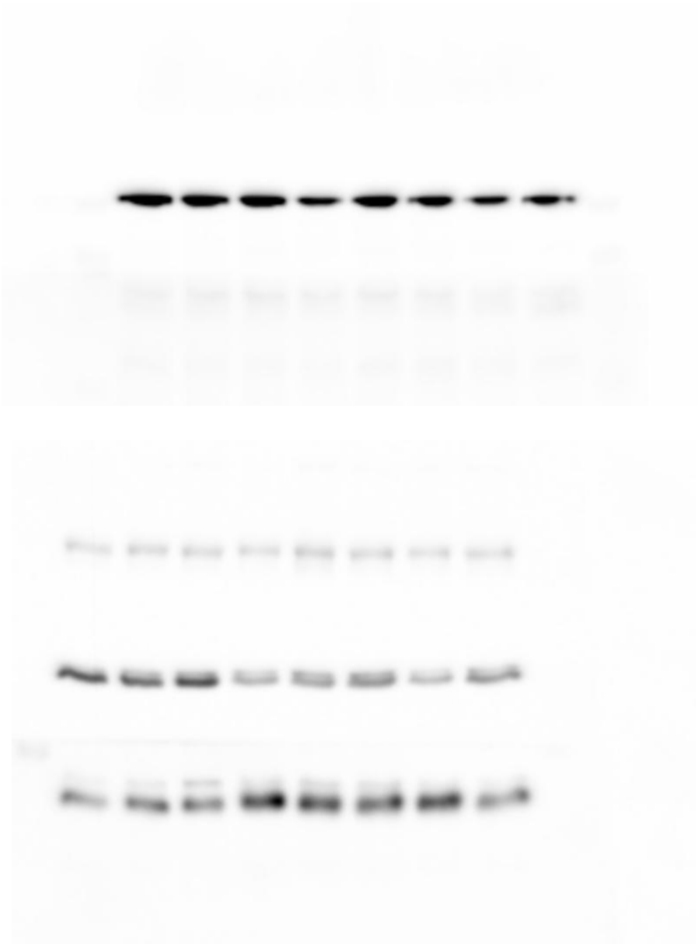

Nrf2

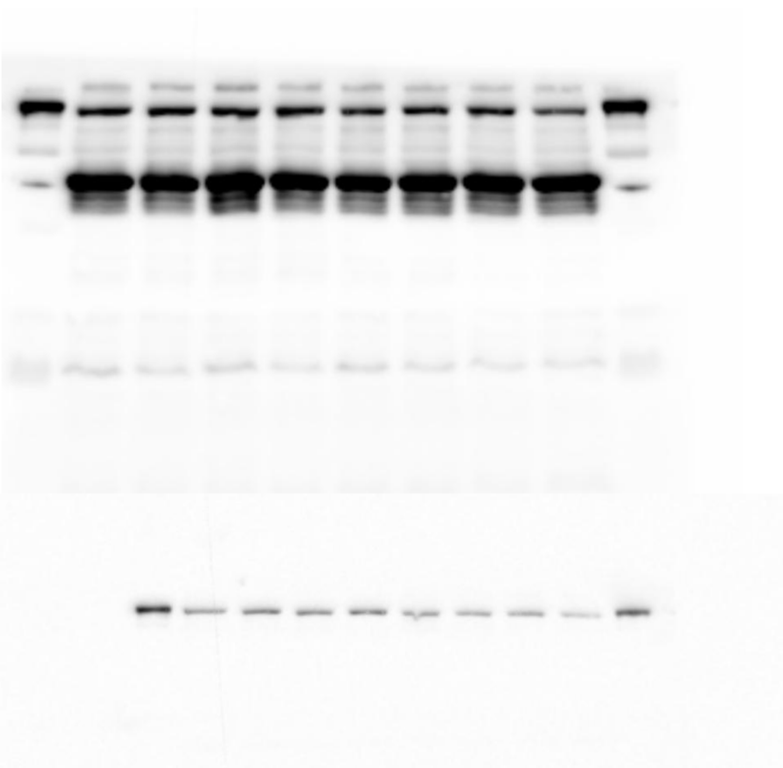

p21

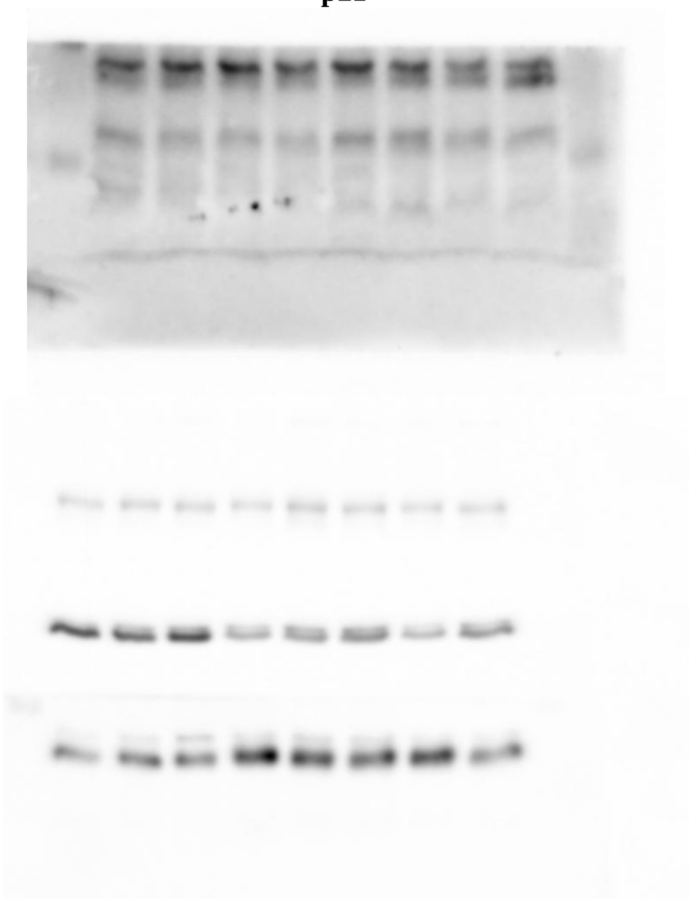

p27

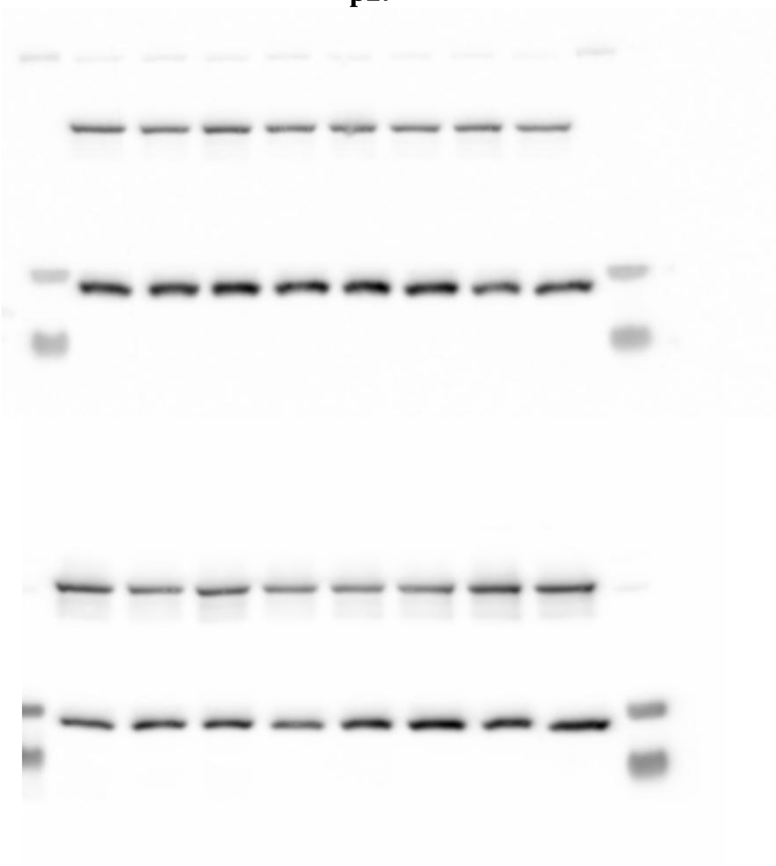

Supplement: Supplementary file 1 — Supplementary file1 (PDF 1860 KB) [file 10571_2025_1610_MOESM1_ESM.pdf]
